# Structural order as a genuine control parameter of dynamics in simple glass formers

**DOI:** 10.1038/s41467-019-13606-3

**Published:** 2019-12-06

**Authors:** Hua Tong, Hajime Tanaka

**Affiliations:** Department of Fundamental Engineering, Institute of Industrial Science, University of Tokyo, 4-6-1 Komaba, Meguro-ku, Tokyo, 153-8505 Japan

**Keywords:** Structure of solids and liquids, Glasses, Glasses, Chemical physics, Structure of solids and liquids

## Abstract

Glass transition is characterised by drastic dynamical slowing down upon cooling, accompanied by growing spatial heterogeneity. Its rationalisation by subtle changes in the liquid structure has been long debated but remains elusive, due to intrinsic difficulty in detecting the underlying complex structural ordering. Here we report that structural order parameter characterising local packing capability can well describe the glassy dynamics not only macroscopically but also microscopically, no matter whether it is driven by temperature or density. A Vogel-Fulcher-Tammann (VFT)-like relation is universally identified between the structural relaxation time and the order parameter for supercooled liquids with isotropic interactions. More importantly, we find such an intriguing VFT-like relation to be statistically valid even at a particle level, between spatially coarse-grained structural order and microscopic particle-level dynamics. Such a unified description of glassy dynamics based solely on structural order is expected to contribute to the ultimate understanding of the long-standing glass-transition problem.

## Abstract (web summary)

The glass-forming materials exhibit dynamical slowing down together with spatial heterogeneity at microscales, but their origin remains debated. Tong and Tanaka show that this phenomenon can be unified based on a structural order parameter capable of detecting subtle ordering in instantaneous liquid states.

## Introduction

Despite the use of glasses for thousands of years, the nature of glass and the glass transition remains probably the deepest and most interesting unsolved problem in condensed matter physics and materials science^1–8^. In contrast to crystallisation whose solidity is a consequence of the emergence of long-range periodic order, the drastic dynamical slowing down towards the glass transition, either by cooling or densification, does not involve obvious change in the structure that is seen by two-point density correlators accessible through diffraction and scattering experiments^4,7^. Therefore, glasses are often considered as the epitome of a completely disordered state of materials. For example, the glass transition is described as a purely dynamical phenomenon in kinetically constrained models^6^. On the other hand, the physical scenarios which posit a growing static order and hence a thermodynamic origin behind the slowing down of glassy dynamics have regained popularity since the discovery of the so-called dynamic heterogeneity in 1990s^2,9–11^. The spatially correlated domains which move significantly faster or slower than the average are proposed to be the long-sought-after cooperatively rearranging regions (CRRs), which is the core concept of the Adam-Gibbs theory of glass transition^12^ and its modern version, i.e., the random first-order transition (RFOT) theory^5,13^. Meanwhile, a growing static correlation length characterising the extent of heterogeneous dynamics is also suggestive of a similarity between glass transition and the critical phenomena^14–17^. However, since the dynamic heterogeneity is not always accessible through static structural variables, the crucial physical mechanism is still not established, namely a quantitative characterisation of the glassy structural order and its link to dynamics. This fact precludes a decisive underpinning to the (thermodynamic) nature of the glass transition. It is also a fundamental question whether the microscopic dynamic heterogeneity can be understood together with macroscopic slowing down on an equal footing within a unified physical picture.

One typical approach to look for key structural features responsible for glassy dynamics is to consider specific physical aspects of the local atomic environment, e.g., free volume, potential energy, and spatial symmetry. The free volume approach^18,19^ and the inherent potential energy based on the potential energy landscape (PEL) formalism^20^ enjoyed early success showing a clear macroscopic correlation (that is, for globally averaged quantities) with dynamics, but not microscopically for particle-level dynamics^21–23^. Therefore, more efforts have been devoted to the identification of locally favoured structures (LFSs) based on symmetry considerations. For instance, icosahedral^24,25^, crystal-like orders^14–16,26,27^, or low-energy topological clusters^28,29^ are identified and suggested as the origin of slow dynamics in different glass-forming liquids, but unfortunately with varying degrees of success^30^. This situation may arise from the system-dependent nature of locally low free-energy configurations^16,27^. Soft vibrational modes are also found to show a clear correlation with relaxations in supercooled liquids^31,32^, but one may still wonder what the geometric features underlying the soft structures are. Another approach developed by Cubuk et al. recently is to define some structural quantity, “softness”, based on machine-learning methods^33,34^. The strong correlation observed between softness and structure relaxation suggests that important structural features are successfully captured by this method. However, softness is defined in a high-dimensional space with more than 100 structure functions, making a clear identification of glassy structural order difficult. For both theoretical and practical purposes, it is desirable to find a simple physical order parameter capturing the structural characteristics of glass-forming liquids.

In this article, we seek a direct quantitative relation between structural order and slow glassy dynamics. The fundamental question of interest is whether the macroscopic slowing down and the microscopic dynamic heterogeneity in apparently different glass formers, either driven by temperature or density, can be understood in a unified manner from a structural perspective. To this end, we construct structural order parameters $X$ detecting sterically favoured structures, namely $\Theta$ in 2D and $\Omega$ in 3D, in the instantaneous liquid states (see Methods section). Unlike the previous structural order parameters defined for inherent structures^23^, our new order parameters are defined for instantaneous structures of a liquid. The crucial point is that such inherent states are never visited in a real liquid under thermal fluctuations, which are intrinsically under a strong influence of anharmonic effects. Surprisingly, we find that the new structural order parameters have a linear scaling relation with the intensive thermodynamic variables such as temperature $T$ and the inverse of density $\rho$, for sixteen different glass formers in their supercooled states. This linear relation initiates around the onset temperature ${T}_{{\rm{on}}}$ of the super-Arrhenius dynamics and continues towards the ideal glass transition point ${T}_{0}$, but is practically ceased by the dynamical glass transition ${T}_{g}$ (here we mention only temperature but the discussions also apply for density). We further establish a direct quantitative relation between structural order parameters $X$ and structure relaxation time ${\tau }_{\alpha }$ in the Vogel-Fulcher-Tammann (VFT) form, indicating that ${\tau }_{\alpha }(T,\rho )$ is a universal function of $X(T,\rho )$ alone [the $(T,\rho )$-dependence of the former is through the $(T,\rho )$-dependence of the latter]. This further suggests the structural/entropic origin of slow glassy dynamics in line with the Adam-Gibbs scenario^12^. More interestingly, based on a nonlocal excitation scenario and a new calculation of microscopic relaxation time, we confirm that such an intimate structure-dynamics correlation is statistically valid even microscopically at a particle level. These findings suggest that the structural order serves a genuine control parameter of dynamics both globally and locally, and the cooperativity of dynamics is a result of the spatial correlation of structural order. Therefore, our results may provide an essential piece in the microscopic theoretical description of the long-standing glass transition problem from a structural perspective.

## Results

**Fig. 1:**
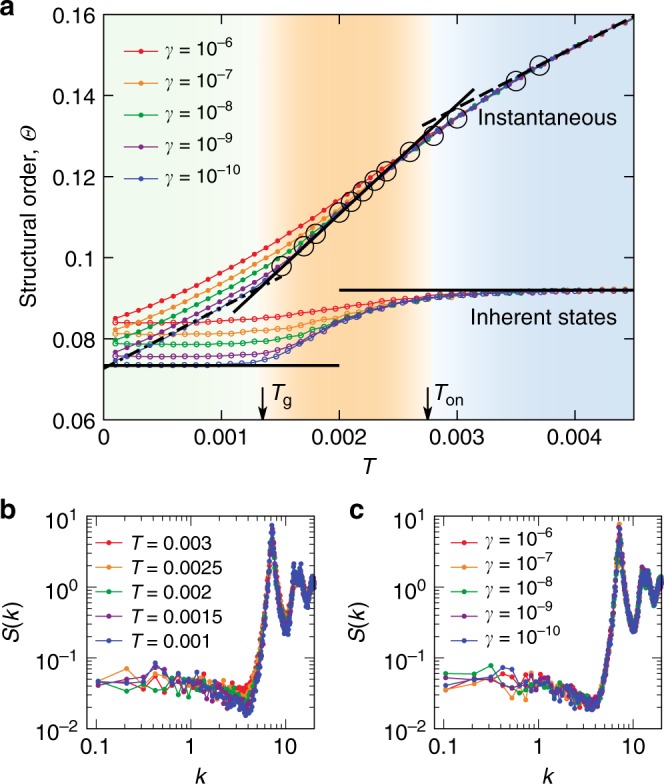
Structure formation during cooling. **a** Evolution of structural order $\Theta$ in instantaneous (filled circles) and inherent states (open circles) in a 2D PM ($\Delta\,=\,13 \%$). Dotted data are shown for different cooling rates $\gamma$. The onset temperature (${T}_{{\rm{on}}}\,=\,0.00276$) and dynamical glass transition temperature for the slowest cooling rate (${T}_{g}\,=\,0.00135$) are indicated, allowing us to separate the simple-liquid (light blue), supercooled (orange), and glass regimes (light green) in a clear manner. For instantaneous states, the temperature dependence of $\Theta$ can be fitted with a linear function in the supercooled regime $(\Theta -{\Theta }_{0})/{\Theta }_{0}\,=\,\kappa (T-{T}_{0})/{T}_{0}$ (solid line), and changes the behaviour at lower (dash-dotted line) or higher temperatures (dashed line). The big open circles indicate equilibrium data from independent simulations. For inherent states, $\Theta$ stays constant in both simple-liquid and glass regimes (horizontal lines). Note that smaller $\Theta$ means higher order. See Table 1 for the fitting parameters and Supplementary Table 1 for the other systems. **b**, **c** Static structure factor $S(k)$ for different temperatures at $\gamma\,=\,1{0}^{-10}$ (panel **b**) and different cooling rates at $T\,=\,0.0015$ (panel **c**). The same colouring scheme in the background is also applied in Fig. 2, for the ease of visualising different temperature regimes.

**Fig. 2:**
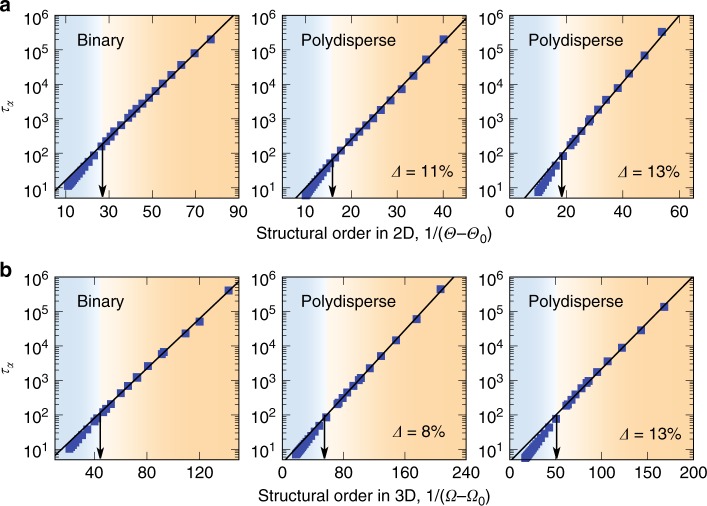
Macroscopic relation between structural order and dynamics. **a** Structure relaxation time ${\tau }_{\alpha }$ as a function of structural order $1/(\Theta -{\Theta }_{0})$ in 2D. **b** ${\tau }_{\alpha }$ as a function $1/(\Omega -{\Omega }_{0})$ in 3D. In the (orange) supercooled regime, the solid lines are commonly the fitting results to the data by the VFT-like relation, ${\tau }_{\alpha }\,=\,{\tau }_{0}\exp [{D}_{2}{X}_{0}/(X-{X}_{0})]$, with $X$ being $\Theta$ or $\Omega$ in 2D and 3D, respectively. See Table 1 for the fitting parameters. The vertical arrows indicate the onset of structural order at ${T}_{{\rm{on}}}$, above which the dynamics does not follow the above VFT-like relation.

**Fig. 3:**
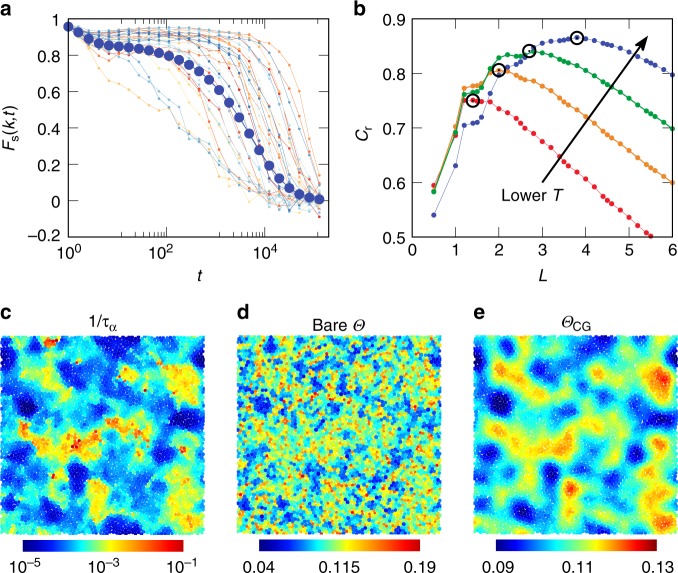
Nonlocal scenario for structure relaxation at a particle level. **a** Self-intermediate scattering function ${F}_{s}(k,t)$ of each particle (background data) and the whole system (dark blue circles) for a 2D PM ($\Delta\,=\,13 \%$) at $T\,=\,0.0018$. **b** Correlation between microscopic relaxation time (${\tau }_{\alpha }$ for each particle, see text for its definition) and structural order as functions of coarse graining length $L$. As indicated by the arrow, different curves correspond to decreasing temperatures, $T\,=\,0.0037$, $0.0028$, $0.0023$, and $0.0018$. The peak positions are indicated by black circles. **c**–**e** For the same state point as panel a, typical snapshots of inverse of microscopic relaxation time $1/{\tau }_{\alpha }$ (panel **c**), bare structural order $\Theta$ (panel **d**), and structural order ${\Theta }_{{\rm{CG}}}$ coarse grained at $L\,=\,3.8$ (panel **e**), where ${C}_{r}$ shows a peak in panel **b**.

**Fig. 4:**
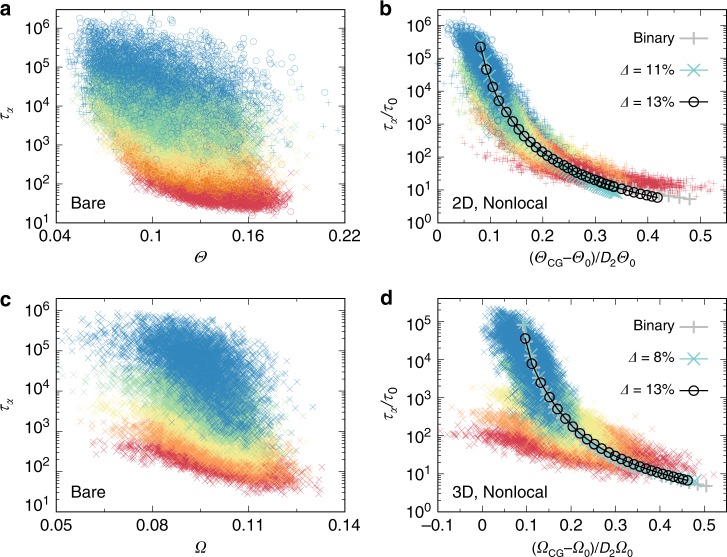
Microscopic relation between structural order and dynamics. **a** Microscopic ${\tau }_{\alpha }$ as a function of bare structural order $\Theta$ for 2D BM and PM ($\Delta\,=\,11 \%$ and $13 \%$) at different temperatures. **b** Corresponding to **a**, microscopic ${\tau }_{\alpha }$ as a function of coarse grained structural order relative to that at ${T}_{0}$, ${\Theta }_{{\rm{CG}}}-{\Theta }_{0}$. On top of the scatter plots, the relations between macroscopic ${\tau }_{\alpha }$ and global structural order are shown together. **c**, **d** The same analysis as in **a** and **b** for 3D systems. For the ease of visualisation, here we show scatter plots only for 3D PM ($\Delta\,=\,8\%$) and put those for BM and PM ($\Delta\,=\,13\%$) in Supplementary Fig. 16. See Table 1 for the parameters used in the plots. The scatter plots are shown for $T\,=\,2.4,2.0,1.7,1.5,1.35\,\times\,1{0}^{-3}$ for 2D BM, $T\,=\,3.0,2.6,2.3,2.0,1.9\,\times\,1{0}^{-3}$ for 2D PM ($\Delta\,=\,11 \%$), and $T\,=\,2.8,2.3,2.0,1.8,1.7,1.51\,\times\,1{0}^{-3}$ for 2D PM ($\Delta\,=\,13 \%$), and $T\,=\,1.25,1.1,1.0,0.9,0.8\,\times\,1{0}^{-3}$ for 3D PM ($\Delta\,=\,8\%$). See Supplementary Fig. 15 for the lengths used for coarse graining. In all cases, the data colour changes from red to blue with a decrease in $T$.

**Fig. 5:**
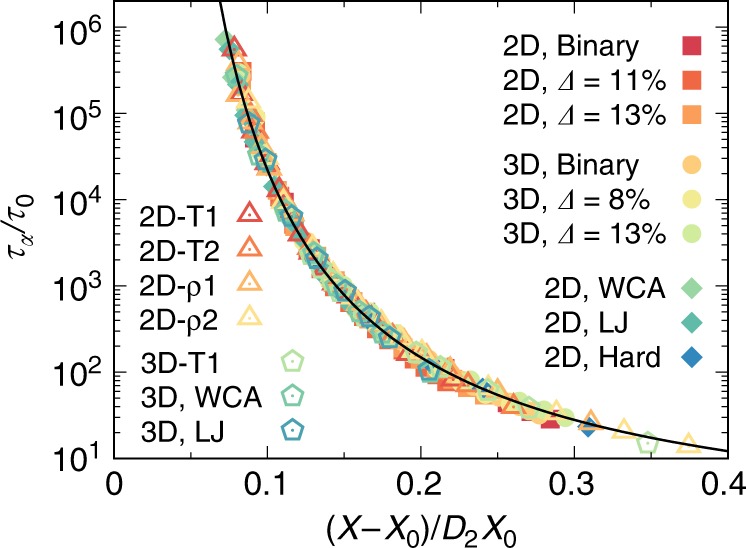
Universal relationship between structural order and dynamics. Macroscopic structure relaxation time ${\tau }_{\alpha }$ as a function of the rescaled structural order $(X-{X}_{0})/{D}_{2}{X}_{0}$ for sixteen different systems in the supercooled regime. Filled symbols: 2D BM and PM ($\Delta\,=\,11\%$ and $13 \%$), and 3D BM and PM ($\Delta =8 \%$ and $13 \%$) with harmonic interactions; and 2D polydisperse mixtures ($\Delta\,=\,13\%$) with WCA, LJ and purely hard interactions. For these nine major systems, both macroscopic and microscopic properties are studied. Open symbols: seven additional systems controlled by temperature $T$ (2D-T1,2 with fixed volume fractions $\phi\,=\,0.88$ and 0.86, respectively; 3D-T1 with $\phi\,=\,0.72$; and 3D WCA and LJ) or density $\rho$ (2D-$\rho$1,2 at fixed temperatures $T\,=\,1{0}^{-3}$ and $1{0}^{-4}$, respectively). If not specified, the interaction is harmonic repulsion. Only macroscopic characteristics are studied for these seven systems. Error bars are comparable to the symbol size and hence not shown. Here, $X$ represents averaged $\Theta$ and $\Omega$ in 2D and 3D, respectively. ${X}_{0}$ is the structural order at the hypothesised ideal glass transition, ${\tau }_{0}$ and ${D}_{2}$ are system-dependent parameters given in Table 1 and Supplementary Table 1. The black curve is given by Eq. (2). Note that the glass transition is driven by density (or pressure) in hard disk and 2D-$\rho$1,2 systems whereas by temperature in the others. The collapse of all data of different systems to a single curve suggests the structural order as a genuine parameter that controls the slowing down of glassy dynamics, no matter whether it is temperature-driven or density-driven.

**Models**. We perform molecular dynamics simulations of sixteen glass formers, covering the degrees of freedom in terms of spatial dimensions, interaction potentials, compositions, and also state points in the phase space controlled by temperature $T$ or density $\rho$ (see Methods section and Supplementary Note 1). Therefore, our study touches upon most of the essential physical factors affecting the glass transition behaviours, whose effects, to the best of our knowledge, have never been described in a unified manner within the same structural perspective. For simplicity, we focus mainly on polydisperse (PM) and binary mixtures (BM) of particles with harmonic potentials in both 2D and 3D as typical examples in the main text (Figs. 1–4). Central results for systems with Lennard-Jones, Weeks-Chandler-Andersen and purely hard interactions are included in Fig. 5. Since liquid structures are complex and temporally fluctuating, in the following, we start from a correlation between globally averaged quantities and then move on to a severer test of the structure-dynamics correlation by investigating their microscopic correspondence.

### Structure ordering towards glass transition

Figure 1a shows the temperature evolution of structural order $\Theta$ in both instantaneous states and the corresponding inherent ones side by side, in 2D PM with $\Delta\,=\,13\%$ for different cooling rates (see Supplementary Figs. 5 and 6 for the other systems). Hereafter instantaneous and inherent structures are always the corresponding ones. Three regimes are clearly identified, i.e., the high-temperature simple-liquid, supercooled, and low-temperature glass states, separated by the onset temperature ${T}_{{\rm{on}}}$ of the super-Arrhenius dynamics (Supplementary Fig. 3) and the dynamical glass transition temperature ${T}_{g}$ (where the system falls out of equilibrium upon cooling). For inherent states, we see no change in the structure in the simple-liquid and glass regimes. It is consistent with the physical picture that the system gets influenced by the underlying PEL only below ${T}_{{\rm{on}}}$, and finally trapped in the basins (metastable states) after a dynamical glass transition at ${T}_{{\rm{g}}}$^3,35^. Accordingly, in the simple-liquid and glass regimes, the evolution of $\Theta$ in instantaneous states is a pure consequence of thermal fluctuations controlled by temperature. Only in the supercooled regime, the glassy structural order grows, leading to a non-trivial linear scaling relation between $\Theta$ and $T$:

1 $$(\Theta -{\Theta }_{0})/{\Theta }_{0}\,=\,\kappa (T-{T}_{0})/{T}_{0}.$$

Here ${\Theta }_{0}$ is the structural order parameter $\Theta$ at the hypothetical ideal glass transition point ${T}_{0}$, which is input from the VFT fitting of ${\tau }_{\alpha }$ (Supplementary Fig. 2), and $\kappa$ is a proportional constant. The same scaling relation is found in 3D for $\Omega$ (Supplementary Fig. 6). Thus, this relation between our structural order parameter ($X\,=\,\Theta$ or $\Omega$) and an intensive thermodynamic variable ($T$ or $1/\rho$) is universal, at least for the systems studied. It is important to emphasise that the structural ordering in the instantaneous states controlling glassy cooperative dynamics is an integrated consequence of not only the structural change in the inherent states but also the thermal fluctuations. Reflecting this feature, as shown in Eq. (1), our order parameter behaves as an effective intensive thermodynamic variable, and thus serves as a genuine control parameter of dynamics. This further leads to the VFT-like relation between the structural relaxation time ${\tau }_{\alpha }(T,\rho )$ and our order parameter $X(T,\rho )$ [see Eq. (2)]. Therefore, even though it might appear as a minor change that we switch from inherent states to instantaneous (thermalized) ones to define the structural order parameter, the inclusion of thermal fluctuations is the key for the order parameter to be a physical quantity that directly controls the slow glassy dynamics (see also Supplementary Figs. 7 and 8 and discussions accordingly). While such structural ordering with a direct link to slow dynamics can be clearly detected by our order parameter, it is invisible through usual two-point correlators, e.g., the static structure factor $S(k)$ (Fig. 1b, c). This fact suggests that many-body correlations are essential to detect the subtle structure ordering in glass-forming liquids^16^.

### Macroscopic relation between structural order and dynamics

Here we seek a direct quantitative relation between our structural order parameter $X$ and the structural relaxation time ${\tau }_{\alpha }$. Figure 2 shows ${\tau }_{\alpha }$ as a function of $X$ for six different glass formers in both 2D and 3D. We find a universal scaling relation in the VFT-like form between ${\tau }_{\alpha }$ and $X$ for all the systems in the (orange) supercooled regime:

2 $${\tau }_{\alpha }\,=\,{\tau }_{0}\exp \left[{D}_{2}{X}_{0}/(X-{X}_{0})\right],$$

where $X$ represents $\Theta$ or $\Omega$ in 2D and 3D, respectively, and ${\tau }_{0}$ and ${D}_{2}$ are fitting parameters. The same scaling relation is also found in systems with different interactions. Actually, Eq. (2) can be deduced from the equation of state based on $X$ [i.e., Eq. (1)] together with the well-established VFT temperature dependence of ${\tau }_{\alpha }$ (Supplementary Fig. 2), and the fittings of the data with Eq. (2) in Fig. 2 further confirm such a relation. The slight deviations above ${T}_{{\rm{on}}}$ (left end) are a consequence of the crossover from the VFT to Arrhenius behaviour around ${T}_{{\rm{on}}}$^16^ (see also Fig. 1a on the crossover). Equation (2) states that the drastic slowing down of glassy dynamics below ${T}_{{\rm{on}}}$ is intrinsically controlled by the structural change of the liquid, no matter whether it is driven by temperature or density, which is well characterised by our order parameter incorporating the influence of thermal fluctuations. As the structure order evolves towards a certain perfect one, ${\tau }_{\alpha }$ steeply increases and tends to diverge towards the ideal glass transition point. This result crucially establishes a direct quantitative relationship between structure and dynamics, thus providing a strong underpinning on the thermodynamic nature of glass transition, more specifically its entropic origin, even though the ideal glass transition is never accessed and thus hypothetical (e.g., ref. ^16^). In this context, it may be interesting to note the similarity between Eq. (2) and the standard Adam-Gibbs relation^12^ ${\tau }_{\alpha }\,=\,{\tau }_{0}\exp (A/T{S}_{{\rm{conf}}})$ ($A$: a constant). This similarity suggests that the configurational entropy ${S}_{{\rm{conf}}}$ is controlled by the degree of structural disorder measured by our order parameter, which provides a direct structural insight into the understanding of configurational entropy. In the Supplementary Figs. 9–11, we have analysed the behaviour of ${S}_{{\rm{conf}}}$ and preliminarily established its connection with our structural order parameter.

### Microscopic relation between structural order and dynamics

Now we move one step forward to seek a microscopic relation between structural order and dynamics. First of all, we point out that a strong microscopic correspondence is essential to establish a concrete structure-dynamics correlation, which relies on the critical many-body information captured by a proper structural order parameter. To this end, it is worth mentioning that many structural quantities, e.g., free volume and potential energy, show a correlation with macroscopic relaxation when globally averaged^18–20^, but do not show obvious correlation with microscopic dynamics at a particle level^21–23^. In order to achieve a meaningful measurement of microscopic relaxation, we have performed simulations in the isoconfigurational ensemble^21,31,36^ and accessed the characteristic structure relaxation of each particle (see Methods section). In Fig. 3a, we plot the self-intermediate scattering function ${F}_{s}(k,t)$ for individual particles together with its global average. Evidently, many particles relax significantly faster or slower than the average, directly indicating strong particle-level heterogeneity in the dynamics. This is also visualised via the contour plot of microscopic relaxation time ${\tau }_{\alpha }^{i}$, defined as ${F}_{s}^{i}(k,{\tau }_{\alpha }^{i})\,=\,{e}^{-1}$ for particle $i$, as shown in Fig. 3c. We note that local ${\tau }_{\alpha }^{i}$ probes the tendency of local structure around particle $i$ to relax, which is directly linked to the free-energy barrier to local structural rearrangements $\Delta {G}^{i}$ as ${\tau }_{\alpha }^{i}\,\sim\,\exp (\Delta {G}^{i}/{k}_{B}T)$. Thus, it provides an effective measurement of particle-level structural relaxation and facilitates a direct comparison between structure and dynamics at a microscopic level, as will be discussed later in Fig. 4. The common approach to identify a microscopic structural feature responsible for the heterogeneous dynamics is to look at the spatial distribution of a specific local structural quantity. Figure 3d shows such a plot of $\Theta$, which exhibits its highly fluctuating character so that only moderate correlation with microscopic ${\tau }_{\alpha }$ can be seen in comparison with Fig. 3c. This weak correlation is a natural consequence of the fact that glassy dynamics is not a purely local caging of particles by their neighbours. The fundamental and unavoidable conclusion is that, if there is a spatial correlation in the structure controlling dynamics, we need to take this fact into account systematically to reveal an intrinsic structure-dynamics relationship. Considering that there exists certain structural order that is correlated over the correlation length of $\xi$, it is reasonable to expect that such structural order would affect dynamics in a correlated manner. More specifically, in a region of high packing capability, particles cannot move independently and have to move cooperatively. If its characteristic size is $\xi$, local structural relaxation should also be correlated over $\xi$. Therefore, to best capture the structure-dynamics correlation, one needs to access the structure information nonlocally over $\xi$. Based on this physical picture, we seek a nonlocal structural approach for microscopic relaxation, through a systematic spatial coarse-graining to detect the correlated nature of structural ordering (see Methods section)^23^. Figure 3b shows the cross-correlation ${C}_{r}$ between microscopic ${\tau }_{\alpha }$ and structural order as functions of coarse-graining length $L$. ${C}_{r}$ significantly increases upon coarse-graining and exhibits a peak of very high values at a temperature-dependent length scale, which is identified as the characteristic static correlation length $\xi$ of the underlying structure (see Supplementary Figs. 13–15 for other systems). We note that such correlation is significantly stronger than that from the structural order in the corresponding inherent state (specifically, here the peak correlation at the lowest temperature reaches $\sim \! 0.87$, which is more than $20 \%$ higher than that based on inherent states, see Supplementary Fig. 8 for a comparison). This fact strongly supports our claim that it is the instantaneous structure rather than the inherent one that controls the particle dynamics. The critical point is that the inherent structure is a state that is never really visited by a system in a liquid state. This fact can naturally be understood by noting that a liquid at finite temperatures inevitably suffers from strong anharmonic effects. So the structural order parameter defined in instantaneous structures (including thermal fluctuations) should be regarded as a true measure of the liquid structure that directly controls dynamics.

The enhancement of structure-dynamics correlation through spatial coarse-graining over $\xi$ is further illustrated by the spatial distribution of coarse-grained order parameter field ${\Theta }_{{\rm{CG}}}$, as shown in Fig. 3e, which displays remarkable resemblance to the one of microscopic ${\tau }_{\alpha }$ in Fig. 3c. This result is a clear illustration that the microscopic relaxation at a particle level is actually controlled by the structure in a nonlocal manner, consistent with the common belief of cooperative relaxations in supercooled liquids^5,7,12,13,37^. A deep link between our structural order parameter and local structure entropy^15^ is discussed in Supplementary Fig. 12 in this context.

We further investigate quantitatively how the microscopic structure relaxation is related to the structural order. In Fig. 4a, c, microscopic ${\tau }_{\alpha }$ of each particle are plotted as functions of bare structural order parameters $\Theta$ and $\Omega$ in 2D and 3D, respectively, for a range of temperatures from slightly above ${T}_{{\rm{on}}}$ to deeply supercooled regime. Roughly, longer ${\tau }_{\alpha }$ is seen for more ordered particles (with smaller values of $\Theta$ or $\Omega$), with an overall tendency of structure ordering and dynamical slowing down upon cooling. However, the correlation is rather weak, as can be seen from largely scattered data.

We then apply spatial coarse-graining to the structural order at its characteristic static correlation length $\xi$ as determined by Fig. 3b (see also Supplementary Figs. 13–15 for the other systems), and replot the data in Fig. 4b, d. Besides, the data sets of macroscopic ${\tau }_{\alpha }$ versus the global structural order are shown on top. Overall, the very much scattered data points in Fig. 4a, c now shrink horizontally. This tendency is much stronger at lower temperatures, giving rise to a stronger structure-dynamics correlation. In 2D, the microscopic data from three different glass formers collapse nicely onto each other and form a universal thin strip, which further coincides with the macroscopic VFT-like relation (Eq. (2)) between structural order and relaxation (Fig. 4b). This is surprising, because the dynamical difference between any two state points is controlled by the intensive thermodynamic variable (here, which is temperature or/and density), whereas the difference between particles in the same state point is controlled by thermal fluctuations; therefore, inherent difference might be expected for these two cases. Here we note that such coincidence is better at lower temperatures: larger deviations in the slope can be observed at higher temperatures. Such deviations are more significant in 3D, where a pronounced coincidence is accessed only at the lowest temperatures under study (Fig. 4d). The physical factor behind this trend is the much shorter static correlation length in 3D than 2D ($\xi\,=\,1\,\sim\,3.1$ in 3D compared to $1.4\ \sim \ 5.1$ in 2D, see Supplementary Fig. 15) at comparable ${\tau }_{\alpha }$. Note that the relation ${\tau }_{\alpha }\,=\,{\tau }_{0}\exp [D{(\xi /{\xi }_{0})}^{d/2}]$, where $d$ is the spatial dimension and $D$ is the so-called fragility index, is established in previous studies including the systems studied here^23^, which is consistent with Ising-like critical^15–17^ or RFOT^5,13^ scenarios of glass transition. This relation suggests a much faster increase of ${\tau }_{\alpha }$, or the activation energy, with the growth of $\xi$ for $d\,=\,3$. Consequently, the domain size of cooperatively relaxing regions is considerably larger even under moderate supercooling for $d\,=\,2$ than $d\,=\,3$, and hence the coarse-graining over this size more effectively brings the self-averaging effect. This means that for $d\,=\,3$, we need deeper supercooling (meaning longer ${\tau }_{\alpha }$) to have the same level of the effect. We also note that the microscopic VFT-like relation between structure and dynamics should be understood in a statistical sense, due to the inherently stochastic nature of thermal fluctuations (see Supplementary Fig. 17).

### Universal relationship between structural order and dynamics

We have so far found a universal linear scaling relation between structural order parameter ($X$) and an intensive thermodynamic variable ($T$ or $1/\rho$) and thus established a universal quantitative VFT-like relation between $X$ and ${\tau }_{\alpha }$ in the supercooled liquid state of simple glass formers, no matter whether it is driven by temperature or density. To highlight this point, we show in Fig. 5 the structure-dynamics relations for all the sixteen systems studied, covering both binary and polydisperse glass formers in different state points (in the $\rho$–$T$ phase space), for 2D and 3D, and for the four types of interaction potentials, all of which collapse nicely onto the VFT-like relation. This strongly suggests the universality of the VFT-like relation between $X$ and ${\tau }_{\alpha }$ at least for systems interacting with isotropic additive interactions, which we call ‘hard-sphere-like’ systems. The link between structure and entropy in this type of systems further indicates a universal thermodynamic nature of the glass transition, in line with the Adam-Gibbs scenario. Furthermore, we establish a nonlocal mechanism for structure relaxation, which facilitates the identification of a microscopic VFT-like relation between spatially coarse-grained structural order and relaxation at a particle level. These findings lead to an intriguing physical picture that unifies the macroscopic slowing down (driven by an intensive thermodynamic variable such as temperature or density) and the microscopic dynamic heterogeneity (induced by thermal fluctuations), which apparently look independent with each other, under a simple structural approach. Since the structural and dynamical information necessary in our methodology can be easily accessed in modern colloidal experiments^38,39^, we call for experimental verification of our findings.

Here, it is worth mentioning the indication of our results on the roles of the attractive and repulsive pair forces in the dynamic behaviours of WCA and LJ liquids. Previously, the same structure as measured by pair correlation functions but very different dynamics were found between the two systems^40,41^. This led to vivid discussions on the mysterious effect of attractive forces on dynamics in supercooled liquids^40–42^. Our characterisations of WCA and LJ systems, however, provide a simple yet appealing solution. We find that the structure measured by our structural order parameter is actually quite different between the two liquids; however, the dynamics is controlled commonly by the structure order parameter following the same physical scenario (Fig. 5). This result clearly indicates the essential significance of many-body correlation in the physical description of supercooled liquids, which is captured by our structural order parameter but missed by pair correlation functions. Considering that such intricate structural ordering at many-body level is also influenced by long-range attraction, this finding suggests a direction for improvement of the standard van der Waals picture of liquids^43^.

The general observation of a VFT-like relation between structural order and relaxation shown in Fig. 5 is surprising, at least at first sight. This is related to the linear scaling relation [see Eq. (1)], to a good approximation, between our structural order parameter and intensive thermodynamic variables in supercooled liquids. Considering that the order parameters we defined closely measure the deviation from the most efficiently packable local structures, with the same spirit in 2D and 3D, the observed linear scaling relation as the first-order approximation might be understandable. However, a strict demonstration is lacking at this moment due to the difficulty associated with the many-body nature of the order parameters. This issue remains for future research. Here we emphasise that such a universal VFT-like relation can only be established based on structural order in the instantaneous states, which is a true measure of the liquid structures including thermal fluctuations really visited at finite temperatures. Moreover, we note that in Eq. (2) the dynamics is controlled only by the structural order parameter $X$ (meaning that temperature or density is implicit), suggesting a purely static picture for slow glassy dynamics, i.e., its thermodynamic origin. The collapse of the data of the sixteen systems in Fig. 5 is also suggestive of hidden similarity among apparently different systems. A possible connection between our observation and the existence of isomorph in simple liquids^42,44,45^, namely a class of equivalent state points in the phase diagram that have the same structure and dynamics in reduced units, is an interesting direction to explore in the near future.

## Discussion

The microscopic relaxation, which we characterise using ${\tau }_{\alpha }$ of individual particles, is found to be nonlocally correlated with the structural order. Such a nonlocal mechanism of structure relaxation is expected to constitute an essential piece for a complete understanding of glass transition, which should be accounted in general in theoretical approaches. The fact that we have the local VFT-like relation between structural order parameter and dynamics only after spatial coarse-graining of the former suggests that the cooperativity of dynamics is a direct consequence of the spatial correlation of structural order. In particular, it provides the physical mechanism connecting a growing length scale with the dynamical slowing down at a particle level (which is not the case in ordinary critical phenomena).

Finally, we note that the structure of hard-sphere glasses is driven by the entropy alone, or the packing effect, since there is intrinsically no energy term in the free energy. We use ‘hard-sphere-like’ to categorise glass formers whose structure ordering is also dominated by the entropy, or the packing effect, which can be captured by our structural order parameter. We stress that many model glass formers with simple isotropic interactions, e.g., those studied in the present work, as well as most of the previous numerical simulation studies, fall into this category^27^. In the absence of obvious density inhomogeneity beyond the particle size, which is the case for these glass formers, the energy term does not play a role in the selection of locally favoured stable structures^23^, although it may take effect as a global constraint as discussed above. Alternatively, we can also understand the structural ordering as a result of steric repulsion at finite temperature. As a counter-example, strong liquids like silicon and water^46^, local structures of which are dominated by the directional bonding, i.e., the energy, are not hard-sphere-like. In those systems, the loosely-packed tetrahedral structure is preferred, and we actually find a negative correlation between $\Omega$ and dynamics (meaning that particles with small $\Omega$ tend to be mobile). Hitherto, we have used the term ‘hard-sphere-like’ in a somewhat loose manner. Similar to the categorisation of a ‘simple liquid’ in the isomorph theory^42,44,45^, which depends on not only the interaction potentials but also the part of the phase diagram of interest, a clear categorisation of ‘hard-sphere-like’ would require further investigations. We note that these two categorisations may not necessarily be the same, although overlap is expected. Such a categorisation is expected to simplify the description of various glass formers, which are apparently different but may follow the same physical scenario that the underlying structural ordering controls slow glassy dynamics through a nonlocal mechanism. Different physical scenarios may also be rationalised accordingly for glass formers that fall out of this categorisation.

## Methods

### Models and simulation methods

We perform molecular dynamics simulations of sixteen hard-sphere-like model glass formers with four types of interactions. In the main text we focus on polydisperse (PM) and binary mixtures (BM) of harmonic particles in both 2D and 3D, and use temperature as the controlling intensive thermodynamic variable. The interaction potential between particles $i$ and $j$ is given as $V({r}_{ij})\,=\,\epsilon \ {(1-{r}_{ij}/{\sigma }_{ij})}^{2}\Theta (1-{r}_{ij}/{\sigma }_{ij})/2$ for ${r}_{ij}\,<\,{\sigma }_{ij}$, where ${r}_{ij}$ is the separation between particles $i$ and $j$, ${\sigma }_{ij}$ is the sum of their radii, and $\Theta (x)$ is the Heaviside step function. These models have been widely studied as canonical glass formers which show behaviours as quasihard particles^47,48^. For the polydisperse case, the particle size is extracted from a Gaussian distribution with polydispersity $\Delta\,=\,\sqrt{\langle {\sigma }^{2}\rangle -{\langle \sigma \rangle }^{2}}/\langle \sigma \rangle$. Two polydispersities are implemented in both 2D and 3D, to introduce different degrees of geometric frustrations against crystallisation to the systems^16^. For the binary case, we mix the equal number of large and small particles, whose diameter ratio is $1.4$. The unit of length is set to the averaged diameter $\langle \sigma \rangle$ and the small particle diameter ${\sigma }_{s}$ in PM and BM cases, respectively. For all cases, the particles have the same mass $m$. The energy, time and temperature are in units of $\epsilon$, $\sqrt{m{\sigma }^{2}/\epsilon }$ ($\sigma$ represents $\langle \sigma \rangle$ or ${\sigma }_{s}$) and $\epsilon /{k}_{{\rm{B}}}$, where ${k}_{{\rm{B}}}$ is the Boltzmann constant. The volume fraction (hence, the density) is fixed at $\phi\,=\,0.91$ in 2D, and $\phi =0.66$ and $\phi =0.67$ for 3D BM and PM cases, respectively, so that the systems have a well-defined inherent state at zero temperature. The total number of particles is $N\,=\,10,000$ for the PM of $\Delta\,=\,11\%$ in 2D and $\Delta\,=\,8\%$ in 3D, and $N\,=\,4096$ for all the rest. We have also studied 2D PM with Lennard-Jones, Weeks-Chandler-Andersen, and purely hard interactions^49^, and the numerical details are given in the Supplementary Note 1.

In addition to the above mentioned nine major systems in which both macroscopic and microscopic properties are characterised (see below), we have studied another seven systems in different conditions only for macroscopic characteristics, as detailed in Supplementary Table 1. Overall, we have accessed the degrees of freedom in terms of spatial dimensions, interactions, compositions, and also regimes in the phase space controlled by temperature or density, and confirmed consistent results.

We employ molecular dynamics (MD) simulations since the focus here is to understand the relationship between structure and glassy dynamics. Swap Monte Carlo method^50^ is attractive in the sense that it allows us to access static information in a more deeply supercooled state, but does not provide realistic dynamical information. Simulations are performed in square boxes for 2D and in cubic boxes for 3D in the $NVT$ ensemble with a Berendsen thermostat^49,51^. We employ periodic boundary conditions. More details on the equilibration and the isoconfigurational ensemble^21,36^ are given in the Supplementary Note 1.

### Characterisations of structure

A set of structural order parameters, $\Theta$ in 2D and $\Omega$ in 3D, is constructed to characterise sterically favoured configurations with high local packing capability in hard-sphere-like glass formers, such as polydisperse and binary mixtures, in a unified manner^23^. They are designed to measure the deviation of a local packing from the perfect arrangement for which neighbouring particles can be most efficiently packed around the central particle. Such a perfect reference is automatically determined for each particle taking into account the particle sizes, the number and arrangement of neighbours, which does not require a prior knowledge of what kind local structure or symmetry would be preferred. Therefore, the advantage of our order parameters lies in their ability to detect exotic amorphous order in an order agnostic manner, which distinguishes them from the common bond orientational order parameters^25,27^.

The critical point here is that the structural order is measured in the instantaneous states at a real liquid temperature. This differs from previous works where the structure is measured in the inherent states at zero temperature^23,34^. Although it might appear as a minor change, it has a very fundamental physical significance that is crucial to establish a quantitative relationship with the dynamics. This fact can be understood from the fact that the inherent structure is a state that is never really visited by a system in a liquid state. In the absence of obvious density inhomogeneity beyond the particle scale, the sterically favoured structures provide more room for particles to move vibrationally through better arrangements and hence higher correlational (or, vibrational) entropy, leading to lower local free energy.

**Fig. 6:**
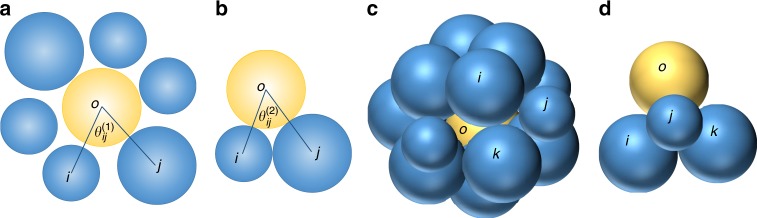
Definition of the structural order parameter. The panels **a** and **b** are for a 2D disk system. **a** A typical local particle configuration consisting of a central particle, $o$, and its $6$ neighbours. We denote two of the neighbours next to each other as $i$ and $j$. **b** The reference configuration corresponding to that in panel a, where the three particles $i$, $j$, and $o$, just touch with each other. We define the central angles of configurations in panels **a** and **b** and denote them as ${\theta }_{ij}^{(1)}$ and ${\theta }_{ij}^{(2)}$, respectively. The panels **c** and **d** are for a 3D sphere system. **c** A typical local particle configuration consisting of a central particle, $o$ and its $14$ neighbours. We denote three of the neighbours next to each other as $i$, $j$, and $k$. **d** The reference configuration corresponding to that in panel **c**, where the four particles, $i$, $j$, $k$, and $o$, just touch with each other. We describe the detailed definition of the structural order parameter for the instantaneous liquid states in the main text.

2D case.—A typical local configuration in 2D disk systems, which consists of a central particle $o$ and its neighbours, is shown in Fig. 6a. All through our analyses, we define the neighbouring particles by using the radical Voronoi tessellation in order to handle the particle size differences^52^. For each pair $\langle ij\rangle$ of neighbouring particles next to each other, we measure the angle between ${{\bf{r}}}_{oi}$ and ${{\bf{r}}}_{oj}$, which we denote as ${\theta }_{ij}^{(1)}$. Such triangles of three neighbouring particles are regarded as fundamental structural units in 2D. In Fig. 6b, we illustrate the corresponding reference configuration of these three particles perfectly just in touch. Here we indicate the central angle as ${\theta }_{ij}^{(2)}$. The structural order parameter for particle $o$ is then defined as

3 $${\Theta }_{o}\,=\,\frac{1}{{N}_{o}}\sum _{\langle ij\rangle }| {\theta }_{ij}^{(1)}-{\theta }_{ij}^{(2)}| ,$$

where ${N}_{o}$ is the number of pairs of neighbours, which is the same as the number of neighbours of particle $o$, and the summation runs over all pairs of neighbours that are next to each other. $\Theta$ is a measure of the deviation from sterically favoured structures, i.e., the deviation of a local packing from the configuration in which neighbouring particles can be most efficiently packed around the central particle. Larger $\Theta$ means more significant deviation from the sterically favoured configuration, and hence more disordered.

3D case. —A typical local configuration in 3D sphere systems, which consists of the central particle $o$ and its neighbours, is shown in Fig. 6c. Note that we identify the neighbour shell by using the radical Voronoi tessellation^52^. Then, we select all sets of three neighbours that form a tetrahedron together with the central particle. We note that these three particles contribute to a vertex of the Voronoi cell of the central particle. We show an example of such selection in Fig. 6c. We denote the lengths of each edge of this tetrahedron as ${r}_{oi}$, ${r}_{oj}$, etc. Similarly to the 2D case, we construct the corresponding reference tetrahedron consisting of the four particles that are perfectly just in touch, as shown in Fig. 6d. We denote the edge lengths as ${\sigma }_{oi}$, ${\sigma }_{oj}$, etc. Then, we quantify the imperfection of a tetrahedron of the original configuration as

4 $${\omega }_{\langle oijk\rangle }\,=\,\frac{{\sum }_{\langle ab\rangle }| {r}_{ab}-{\sigma }_{ab}| }{{\sum }_{\langle ab\rangle }{\sigma }_{ab}}.$$

Here $\langle ab\rangle$ runs over the six edges of the tetrahedron $\langle oijk\rangle$. Finally, we obtain the structural order parameter of particle $o$ as

5 $${\Omega }_{o}\,=\,\frac{1}{{N}_{o}^{{\rm{tetra}}}}\sum _{\langle oijk\rangle }{\omega }_{\langle oijk\rangle },$$

where ${N}_{o}^{{\rm{tetra}}}$ is the total number of tetrahedra including the central particle $o$ as a member and the summation runs over all these tetrahedra. Similarly to $\Theta$ for 2D, $\Omega$ is a measure of the deviation from the sterically favoured configuration, i.e., the deviation of a local packing from the most efficiently packable configuration around the central particle. Larger $\Omega$ means more significant deviation and more disordered.

**Table 1:** Fitting parameters.

	${\Theta }_{0}$ or ${\Omega }_{0}$	${T}_{0}(1{0}^{-4})$ or ${\rho }_{0}$	${\tau }_{0}$	$D$	${D}_{2}$
2D, harmonic, binary	0.0892	6.47	3.95	8.66	1.59
2D, harmonic, $\Delta\,=\,11 \%$	0.0735	9.50	0.677	10.9	4.05
2D, harmonic, $\Delta =13 \%$	0.0784	8.02	1.57	10.1	2.64
3D, harmonic, binary	0.0907	2.24	3.09	15.0	0.889
3D, harmonic, $\Delta =8 \%$	0.0884	5.21	3.84	5.33	0.629
3D, harmonic, $\Delta =13 \%$	0.0882	4.67	2.81	6.70	0.710
2D, WCA, $\Delta =13 \%$	0.0822	608.0	0.352	19.1	2.14
2D, LJ, $\Delta =13 \%$	0.0775	1300.0	0.169	13.3	2.82
2D, hard disk, $\Delta =13 \%$	0.0829	1.01	0.205	0.301	2.04

Here we list the fitting parameters used in the plots according to Eqs. (1) and (2) and in the VFT fitting of the $\alpha$ relaxation time (Supplementary Fig. 2) for nine major systems under study. The glass transition of hard disk systems is driven by density $\rho$ (or pressure) increase, whereas by temperature $T$ decrease for the rest. Note that the coefficient in Eq. (1) can be deduced as $\kappa\,=\,{D}_{2}/D$. Fitting parameters for additional seven systems used in Fig. 5 are given in Supplementary Table 1

The coarse-graining of order parameter $X$ (here $X$ stands for $\Theta$ or $\Omega$) for particle $i$ is calculated by taking its average over all particles within a distance $L$: ${\overline{X}}_{i}(L)\,=\,{\sum }_{j}{X}_{j}P(| {{\bf{r}}}_{j}-{{\bf{r}}}_{i}| )/{\sum }_{j}P(| {{\bf{r}}}_{j}-{{\bf{r}}}_{i}| )$. An exponential core $P(x)\,=\,\exp (-x/L)$ is employed by assuming that the influence of the local structure on the dynamics decays exponentially in space. We emphasise that an order parameter that is able to capture the important many-body correlation is essential for revealing the structure-dynamics correlation, and the spatial coarse-graining is purely a static operation to uncover it.

### Characterisations of dynamics

The dynamics in 2D is characterised using relative positions ${\underline{{\bf{r}}}}_{j}(t)\,=\,{{\bf{r}}}_{j}(t)-{\sum }_{k}{{\bf{r}}}_{k}(t)/{n}_{j}$, where the summation runs over all neighbours of particle $j$. This removes the long-wavelength vibrational motions, known as Mermin-Wagner fluctuations, which are not relevant to structure relaxations^53–56^, and recovers general features of glassy dynamics as in 3D (by using the original positions). To be simple, notions of the original positions are used in the following. We measure the self-intermediate scattering function ${F}_{s}(k,t)\,=\,\langle {\sum }_{j}\exp (i{\bf{k}}\cdot [{{\bf{r}}}_{j}(t)-{{\bf{r}}}_{j}(0)])/N\rangle$, where $k\,=\,| {\bf{k}}|$ takes values at the first peak of the static structure factor and $\langle \cdot \rangle$ denotes time average. The macroscopic structure relaxation time ${\tau }_{\alpha }$ is defined by ${F}_{s}(k,{\tau }_{\alpha })\,=\,{e}^{-1}$. Detailed results are shown in Supplementary Figs. 1–4. Based on the macroscopic ${\tau }_{\alpha }$, we employ the VFT fitting to extract the ideal glass transition temperature ${T}_{0}$. The onset temperature of slow glassy dynamics ${T}_{{\rm{on}}}$ is defined as a temperature where the temperature dependence of ${\tau }_{\alpha }$ switches from Arrhenius to non-Arrhenius behaviour. The dynamical glass transition temperature ${T}_{g}$ is defined as a temperature where the system falls out of equilibrium upon cooling, and thus it depends on the cooling rate. In Fig. 1, we indicate ${T}_{g}$ for the slowest cooling rate where ${\tau }_{\alpha }\,=\,1{0}^{6}$ for simplicity.

For the measurement of microscopic relaxation, we perform simulations in the isoconfigurational ensemble^21,36^. The important point here is that, unlike previous studies, the instantaneous states thermalized at fast-$\beta$ time scale (hence the structure is not relaxed) are defined as the initial configurations ($t=0$) of the isoconfigurational ensemble, and used for characterisations of structure and dynamics. Similar treatment can be found in ref. ^57^. We define ${F}_{s}^{j}(k,t)\,=\,{\langle \exp (i{\bf{k}}\cdot [{{\bf{r}}}_{j}(t)-{{\bf{r}}}_{j}(0)])\rangle }_{{\rm{iso}}}$ for particle $j$, and the microscopic ${\tau }_{\alpha }$ is deduced from ${F}_{s}^{j}(k,{\tau }_{\alpha }^{j})\,=\,{e}^{-1}$. Here ${\langle \cdot \rangle }_{{\rm{iso}}}$ stands for the isoconfigurational average.

### Correlation between structure and dynamics

We emply the Spearman’s rank correlation coefficient to quantify the correlation between structure and microscopic dynamics, which is a sensitive measurement of the monotonic relationship between two variables without floppy parameters^23,58^. First, we sort the particles in terms of the structural order (the microscopic ${\tau }_{\alpha }$) with the disordered (the mobile) ones in front, and assign the ranks ${O}_{i}$ (${Q}_{i}$) to each particle $i$. The correlation is then calculated as ${C}_{r}\,=\,1-6{\sum }_{i}{({O}_{i}-{Q}_{i})}^{2}/N({N}^{2}-1)$. ${C}_{r}=1$ indicates a perfect monotonic relation, whereas ${C}_{r}=0$ indicates the absence of the correlation.

## Supplementary information

Supplementary Information

## Data Availability

The data that support the findings of this study are available from the corresponding author upon reasonable request.

## Code availability

The codes that are used to generate results in the paper are available from the corresponding author upon reasonable request.
