# Supplementary material for: Structural order as a genuine control parameter of dynamics in simple glass formers
Source: Nat Commun. 2019 Dec 6;10:5596. doi: 10.1038/s41467-019-13606-3 (PMC6898187; doi:10.1038/s41467-019-13606-3)
Supplement: Supplementary file 1 — Supplementary Information [file 41467_2019_13606_MOESM1_ESM.pdf]

Supplementary Information for "Structural order as a genuine control parameter of dynamics in simple glass formers"

Tong and Tanaka

SUPPLEMENTARY NOTE 1. DETAILS OF SIMULATION METHODS

We have performed molecular dynamics simulations in square boxes in 2D and cubic boxes in 3D, with periodic boundary conditions. Four types of interaction potentials are considered, including harmonic, Lennard-Jones (LJ), Weeks-Chandler-Andersen (WCA), and purely hard interactions. For the former three, the simulations are carried out using velocity Verlet algorithms in the NVT ensemble with a Berendsen thermostat [1, 2], whereas the event-driven molecular dynamics is used for the fourth [3]. We study both binary and continuously polydisperse systems for the harmonic systems and only polydisperse systems for the rest. In total, sixteen different glass formers are studied, among which, for the nine listed in Table 1 of the main text, we characterize both macroscopic and microscopic properties, and for the other seven listed in Supplementary Table 1 we characterize only the macroscopic properties. Therefore, we have accessed the degrees of freedom in terms of spatial dimensions, interactions, compositions, and also regimes in the phase space controlled by temperature or density, and confirmed consistent results. Although we have not studied all combinations of these degrees of freedom exhaustively, generality is expected for what we call "hard-sphere-like" systems. More discussion is given accordingly in the text.

Three sets of simulations are carried out to obtain results shown in the paper: (1) continuous cooling from a high-temperature liquid state to glass state, to mimics the experimental glass transition process; (2) long-time simulations at constant temperatures, to access structural and dynamic properties in thermal equilibrium; (3) isoconfigurational simulations [4, 5], to access microscopic dynamical information. In the following, we first give the simulation details for the harmonic systems. Then, we describe the systems with LJ, WCA, and purely hard interactions, with slightly different settings in simulation details.

Harmonic systems. For all cases, the systems are first equilibrated at high temperature (where $\tau_\alpha < 10$) for $t = 1000$. For the continuous cooling simulations, the systems are then cooled down to $T = 10^{-4}$ in 2D and $T = 2 \times 10^{-5}$ in 3D at a constant cooling rate $\gamma = dT/dt$. For the equilibrium simulations with which we calculate the self-intermediate scattering functions on the fly, the systems are cooled down to target temperatures within a time scale of several tens of the structural relaxation time τ_α and then equilibrated for at least several tens of τ_α before the production sampling. For the isoconfigurational ensemble [4, 5] with which we calculate the microscopic structure relaxation time, the systems are cooled down to target temperatures within around $30\tau_\alpha$ and then equilibrated for another $30\tau_\alpha$. After the equilibration run, we quench the system to the nearest inherent structure by using the FIRE algorithm [6] to remove the thermally-excited random displacements. Using this inherent structure as the initial configuration, 100 trajectories are simulated with different momenta assigned randomly from the appropriate Maxwell-Boltzmann distribution. Results of selected state points using 200 trajectories confirm good convergence in a statistical sense. For the 2D polydisperse system with $\Delta = 11\%$ and 3D polydisperse system with $\Delta = 8\%$, 20 initial configurations are used in the calculations to ensure good statistics; for the rest, 30 initial configurations are used. Because of the limitation of the computational power, for the state point of the lowest temperatures under study, 10 initial configurations are used. We set the target temperatures covering a wide range of τ_α (approximately, $\tau_\alpha \in [30, 10^5]$ in 2D and $[100, 10^5]$ in 3D).

Lennard-Jones and Weeks-Chandler-Andersen systems. We have investigated the Weeks-Chandler-Andersen (WCA) and Lennard-Jones (LJ) systems and confirmed the generality of our findings. The interaction potential between particles i and j is

$$V(r_{ij}) = 4\epsilon \left[\left(\frac{\sigma_{ij}}{r_{ij}} \right)^{12} - \left(\frac{\sigma_{ij}}{r_{ij}} \right)^{6} \right] + f(r_{ij}), \tag{1}$$

when $r_{ij}/\sigma_{ij} < R_c$ and zero otherwise, where r_{ij} is the particle separation, σ_{ij} is the sum of the particle radii, and $f(r_{ij})$ guarantees that the potential and its first derivative are zero at $r_{ij} = R_c\sigma_{ij}$. Here $R_c = 2^{1/6}$ and 2.5 correspond to WCA and LJ systems, respectively. For 2D polydisperse systems ($N = 4096$ particles and particle size polydispersity $\Delta = 13\%$), both macroscopic and microscopic properties are characterized. We set the number density $\rho = 0.89$, so that both WCA and LJ systems have a positive pressure at zero temperature. The simulation methods are the same as soft repulsive systems, except that a smaller time step is used to ensure a good convergence of numerical integration. Without loss of generality, we simulate three initial states in the isoconfigurational ensemble. For 3D systems as listed in Supplementary Table 1, only the macroscopic properties are characterized.

In Methods of the main text, we have defined the structural order parameters for hard-sphere-like systems, for which particles have a well-defined surface, as given by the range of the repulsive interaction. In such systems, it is clear that the main driving force for structural ordering comes from packing and excluded volume effects, which are

Supplementary Table 1: **Fitting parameters.** Here we list the fitting parameters for seven additional systems used in the plot of Fig. 5 of the main text. These are polydisperse mixtures of particles with polydispersity $\Delta = 13\%$, for which only macroscopic properties are characterized. The glass transition of 2D-ρ1,2 systems is driven by density ρ (or pressure) increase, whereas by temperature T decrease for the rest.

Abbreviation	Description	Θ_0 or Ω_0	$T_0(10^{-4})$	τ_0	D	D_2
2D-T1	2D, harmonic, $\phi = 0.88$	0.0804	4.94	2.10	13.6	2.50
2D-T2	2D, harmonic, $\phi = 0.86$	0.0818	2.96	2.97	18.8	2.24
3D-T1	3D, harmonic, $\phi = 0.72$	0.0814	11.3	2.93	2.81	0.745
3D, WCA	3D, WCA, $\rho = 0.977$	0.0770	181.0	0.091	6.79	1.20
3D, LJ	3D, LJ, $\rho = 0.977$	0.0758	345.0	0.106	3.40	1.15
		Θ_0	ρ_0	τ_0	D	D_2
2D-ρ1	2D, harmonic, $T = 10^{-3}$	0.0814	1.14	2.92	0.430	2.33
2D-ρ2	2D, harmonic, $T = 10^{-4}$	0.0850	1.04	14.4	0.313	1.85

captured by our order parameters. In the case of the LJ potential which has a long-ranged attractive tail, it is not immediately clear what should be the exact form of a structure descriptor in the same spirit as ours (see Eq. (3)-(5) in the main text). The situation can be complex, considering the recent studies which suggest a nonperturbative effect of the attractive forces [7, 8]. However, it has been shown that an LJ liquid at typical condensed-phase state points can be categorized as a simple liquid in the sense that the intermolecular interactions may be ignored beyond the first coordination shell [9]. This means that its structure is dominated by what may be termed "packing effects". Therefore, it is reasonable to expect that our structural order parameters characterizing the local packing capability also work in LJ systems. Motivated by the fact that, over a wide range of density in the typical condensed phase, the pair correlation functions $g(r)$ of WCA and LJ systems resemble each other [7], we define the effective radius of an LJ particle at the minimum of the interaction potential. So $\sigma_{i,\mathrm{eff}} = 2^{1/6}\sigma_i$ for both WCA and LJ systems. It is also possible to define the effective radius on the repulsive side of the potential at which the potential energy with respect to the minimum equals the thermal energy or to pick up the position of the first peak of $g(r/\langle\sigma\rangle)$ at R_1 and set $\sigma_{i,\mathrm{eff}} = R_1\sigma_i$. We find that the values of Θ are quite insensitive to these choices because Θ measures orientational rather than translational correlations. Therefore, we use $\sigma_{i,\mathrm{eff}} = 2^{1/6}\sigma_i$ in this study.

Hard disk systems. We have also investigated 2D polydisperse hard disks systems and confirmed the generality of our findings. The mixture of $N = 10000$ disks is simulated with event-driven molecular dynamics using the DynamO package [3]. Similar to the other polydisperse systems studied in this work, the particle size is extracted from a Gaussian distribution with polydispersity $\Delta = 11\%$, for which no transition to a hexatic phase is observed. All particles have the same mass. The unit of length is set by the average disk diameter $\langle\sigma\rangle$ and the time scales are reported using the event-driven unit. All simulations are run at fixed densities $\rho = N/L^2$ in square boxes. After the equilibration runs, we generate the isoconfigurational ensemble from the equilibrated configurations. Without loss of generality, we have simulated three independent initial states at each density, and for each initial state, 100 trajectories are simulated.

SUPPLEMENTARY NOTE 2. ADDITIONAL RESULTS IN HARMONIC SYSTEMS

1. Analysis of dynamics. Here we characterize the structure relaxation in both 2D and 3D glass-forming liquids [2]. In 2D case, we use the position of particle j relative to its n_j neighbouring particles l, $\underline{\mathbf{r}}_j(t) = \mathbf{r}_j(t) - \sum_l \mathbf{r}_l(t)/n_j$, to characterize the dynamics, which helps to remove the long-wavelength Mermin-Wagner fluctuations [15–17]. In Supplementary Figure 1, we shown the temperature dependence of self-intermediate scattering functions $F_s(k,t)$ in both 2D and 3D, where typical features of glassy dynamics, i.e. the two-step relaxation, are generally observed.

The structure relaxation time τ_α is measured from the time decay of the self-intermediate scattering function: $F_s(k,\tau_\alpha) = e^{-1}$. Supplementary Figure 2 shows the temperature dependence of τ_α for the six major systems under study. The drastic dynamical slowing down is well described by the Vogel-Fulcher-Tammann (VFT) relation $\tau_\alpha = \tau_0 \exp[DT_0/(T-T_0)]$, which suggests a divergence of τ_α at the ideal glass transition temperature T_0. The fitting parameters are listed in Table 1 of the main text.

We may also plot τ_α as a function of the inverse of temperature $1/T$, as shown in Supplementary Figure 3, from which a crossover from the Arrhenius to non-Arrhenius behaviours is identified as the onset temperature $T_{\rm on}$ of sluggish glassy dynamics. The appearance of the non-Arrhenius behaviour suggests a growth of the activation energy, which is a clear indication of cooperative dynamics typical for the so-called "fragile" glass-forming liquids. From the potential energy landscape (PEL) formalism, $T_{\rm on}$ also signals the onset of a significant influence from the underlying inherent structures and a change in the manner of exploration of the PEL [18].

Supplementary Figure 4 further shows power-law fittings of the temperature dependence of τ_α, as predicted by the

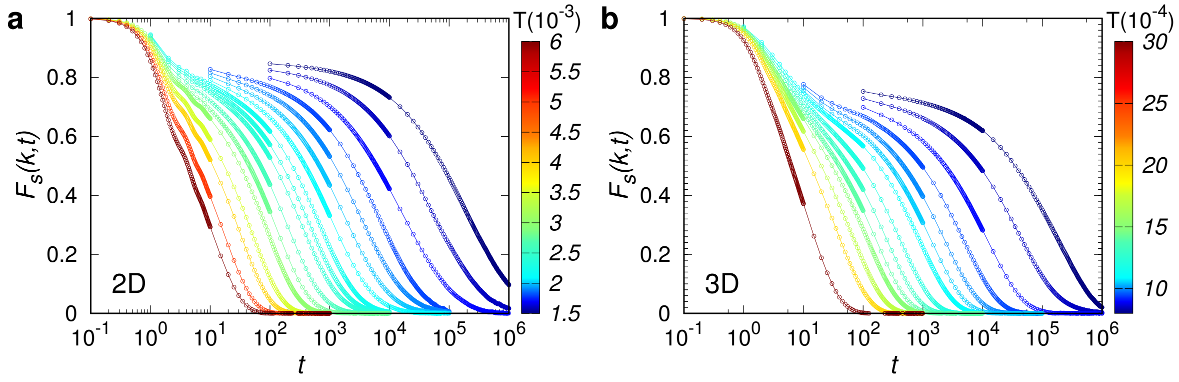

Supplementary Figure 1: **Self-intermediate scattering function in harmonic systems.** Typical temperature dependence of $F_s(k,t)$ for 2D polydisperse systems with $\Delta = 13\%$ (**a**) and 3D polydisperse systems with $\Delta = 8\%$ (**b**). Note that $F_s(k,t)$ is calculated using relative positions in 2D.

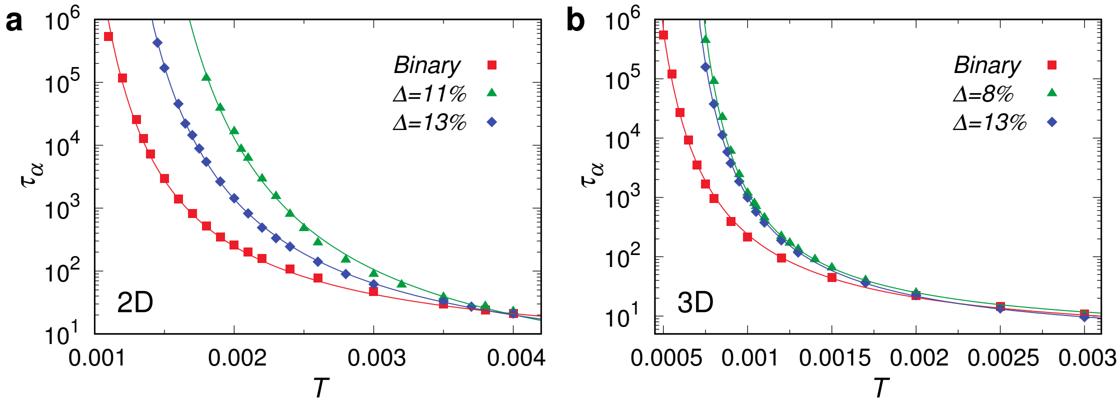

Supplementary Figure 2: **VFT fittings of the temperature dependence of τ_α in harmonic systems.** Temperature dependence of τ_α in 2D disk systems (**a**) and 3D sphere systems (**b**). The solid lines are fittings according to the VFT law $\tau_\alpha = \tau_0 \exp[DT_0/(T-T_0)]$, from which we extract the ideal glass transition temperature T_0.

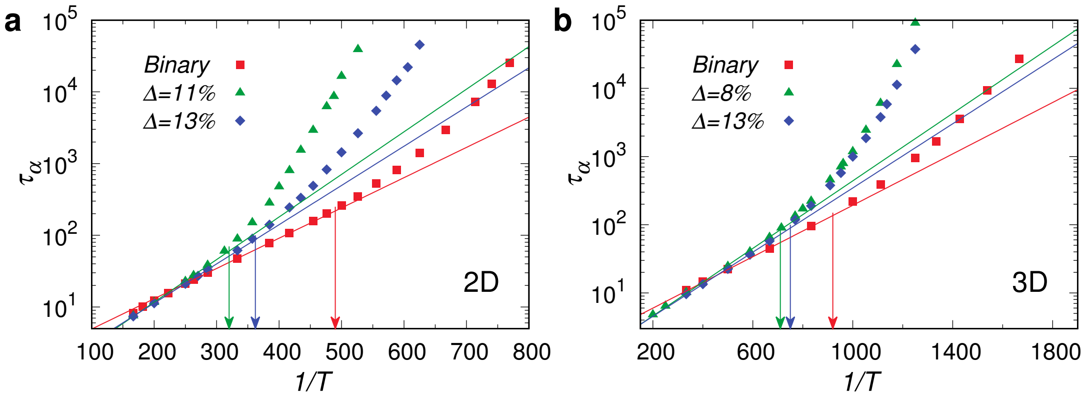

Supplementary Figure 3: **Arrhenius to non-Arrhenius crossover in the temperature dependence of τ_α in harmonic systems.** τ_α as functions of $1/T$ in 2D disk systems (**a**) and 3D sphere systems (**b**). The solid lines show fittings to the high-temperature data, which follows the Arrhenius behaviour $\tau_\alpha \sim \exp(\Delta E/T)$. The crossover where τ_α starts to deviate from the Arrhenius behaviour is identified as the onset temperature of sluggish glassy dynamics,$T_{\rm on}$, as indicated by the arrows.

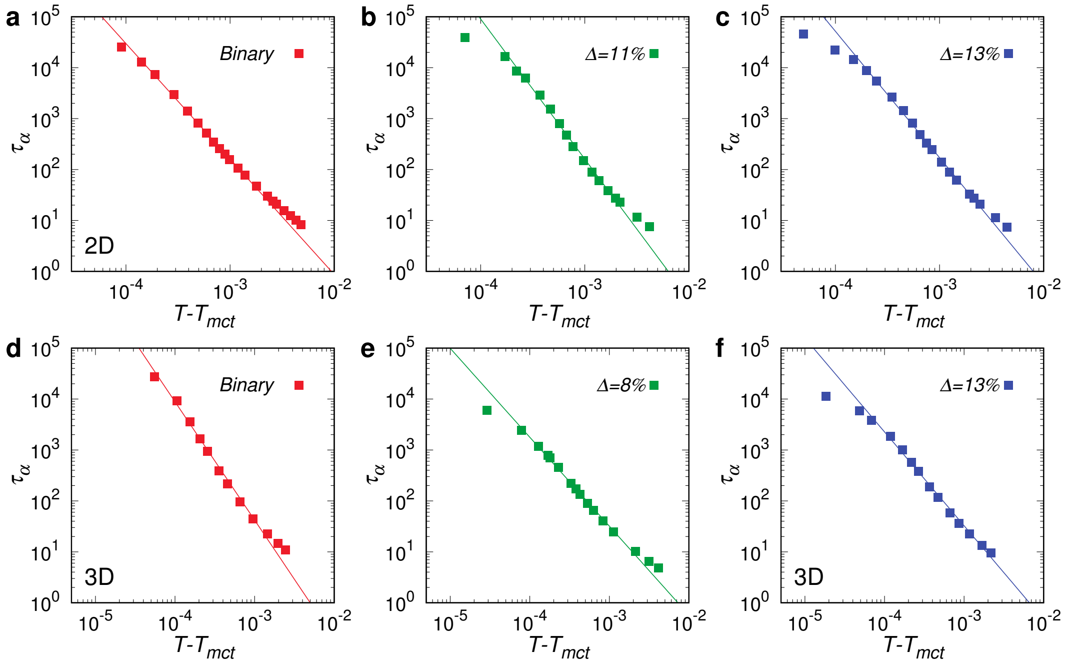

Supplementary Figure 4: **Power-law fittings of the temperature dependence of τ_α in harmonic systems.** τ_α as functions of $T - T_{\rm mct}$ in (**a-c**) 2D disk systems and (**d-f**) 3D sphere systems. The solid lines show power-law fittings according to the mode-coupling theory (MCT) $\tau_\alpha \sim (T - T_{\rm mct})^{-\delta}$, with $T_{\rm mct}$ being the MCT critical temperature. For the data in panels a-f, $T_{\rm mct}$ is determined as $T_{\rm mct} = 0.00121, 0.00183, 0.00155, 0.00054, 0.00087$, and 0.00083, respectively.

mode-coupling theory (MCT). MCT captures the initial stage of slowing down upon cooling and predicts a divergence of τ_α toward $T_{\rm mct}$, which however does not take place in reality. As seen from Supplementary Figure 4, the growth of τ_α eventually deviates from the MCT prediction at low temperatures. Nevertheless, $T_{\rm mct}$ provides the reference information on the temperature range, or the degree of supercooling, at which one is looking. This analysis suggests that our simulations cover the range of temperature down to around $T_{\rm mct}$. We note that the determination of $T_{\rm mct}$ from power-law fittings is purely empirical and not rigorous. Within numerical precision, our result that $T_{\rm mct} = 5.4 \times 10^{-4}$ for the 3D binary system is consistent with Ref. [19], where $T_{\rm mct} = 5.2 \times 10^{-4}$ was found. The slight difference may originate from the fact that big and small particles are analyzed separately in Ref. [19] but all together in current work.

2. Glassy structure formation towards glass transition. Corresponding to Fig. 1a in the main text, in Supplementary Figs. 5 and 6, we show the glassy structure formation for the rest five systems in 2D and 3D, respectively. Universal behaviours are observed, including linear temperature dependence of structural order of instantaneous states in the supercooled regime, and a constant structural order of inherent states in both the high-temperature simple liquid and low-temperature glass regimes. This confirms the general validity of our results.

3. A close comparison of instantaneous and inherent states. Corresponding to Fig. 1a of the main text, here we further unveil the role of structural order in glassy dynamics by a close comparison of instantaneous and inherent states. In Supplementary Figure 7a, we show the average number of neighbour change N_c between the corresponding configurations in instantaneous and inherent states. Here the neighbouring particles are defined by the radical Voronoi tessellation [20]. We can see that N_c turns to increase rapidly above T_g, which is a clear indication that local structural order is destructed under thermal noise. Supplementary Figure 7b further shows that the more disordered structures are less resistive to destruction due to thermal noise. Since Θ can be either bigger or smaller than Θ_{IS}, it is essential to note that the thermal fluctuation may either increase or decrease local structural order in instantaneous configurations, and hence its role is highly nontrivial in supercooled liquids. The substantial structural difference between instantaneous and inherent states in the supercooled regime (see Fig. 1a in the main text) indicates that the inherent structures do not properly reflect the real liquid structures, which are under intrinsic influence of thermal fluctuation (or, entropy). Thus, we argue that it is the instantaneous state rather than the inherent one that we should look at for a quantitative relation between structure and dynamics. We note that this fact has not been taken seriously in many previous works.

We further illustrate this point via a comparison of structure-dynamics correlations based on instantaneous (thermalized) and inherent states. As shown in Supplementary Figure 8 for a 2D polydisperse system ($\Delta = 13\%$) at $T = 0.0018$, the peak structure-dynamics correlation is increased by more than 20% and reaches $C_r = 87\%$ by starting from instantaneous (thermalized) states. Such a high structure-dynamics correlation is unprecedented and crucial to the determination of the quantitative VFT-like relation. Therefore, even though it might appear as a minor change that we switch from inherent structure to instantaneous (thermalized) structure to define the structural order parameter, it has a very fundamental physical significance. The important point is that the inherent structure is a state that is never visited by a system in the liquid state. So the structural order parameter defined for instantaneous structures should be regarded as a true measure of the liquid structure. Because of this feature, this order parameter can have the role of a genuine control parameter, or an effective thermodynamic intensive variable.

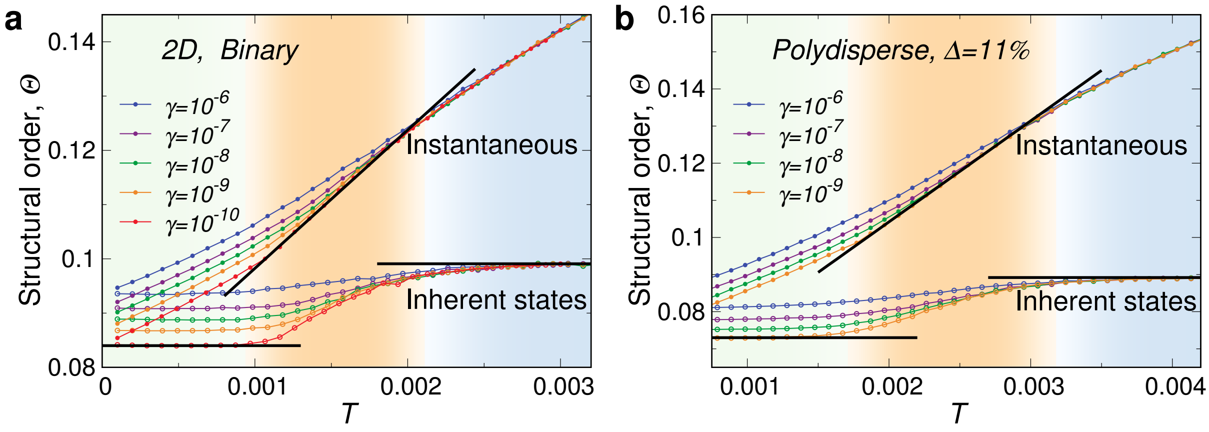

Supplementary Figure 5: **Structure formation during cooling in 2D harmonic systems.** Corresponding to Fig. 1a in the main text, evolution of structural order Θ in instantaneous (filled circles) and corresponding inherent states (open circles) in 2D binary systems (**a**) and 2D polydisperse systems ($\Delta = 11\%$) (**b**). Data are shown for different cooling rate γ. For instantaneous states, the temperature dependence of Θ can be fitted with a linear function in the supercooled regime $(\Theta - \Theta_0)/\Theta_0 = \kappa(T - T_0)/T_0$ (solid line). For inherent states, Θ stays constant in both simple-liquid and glass regimes (horizontal lines).

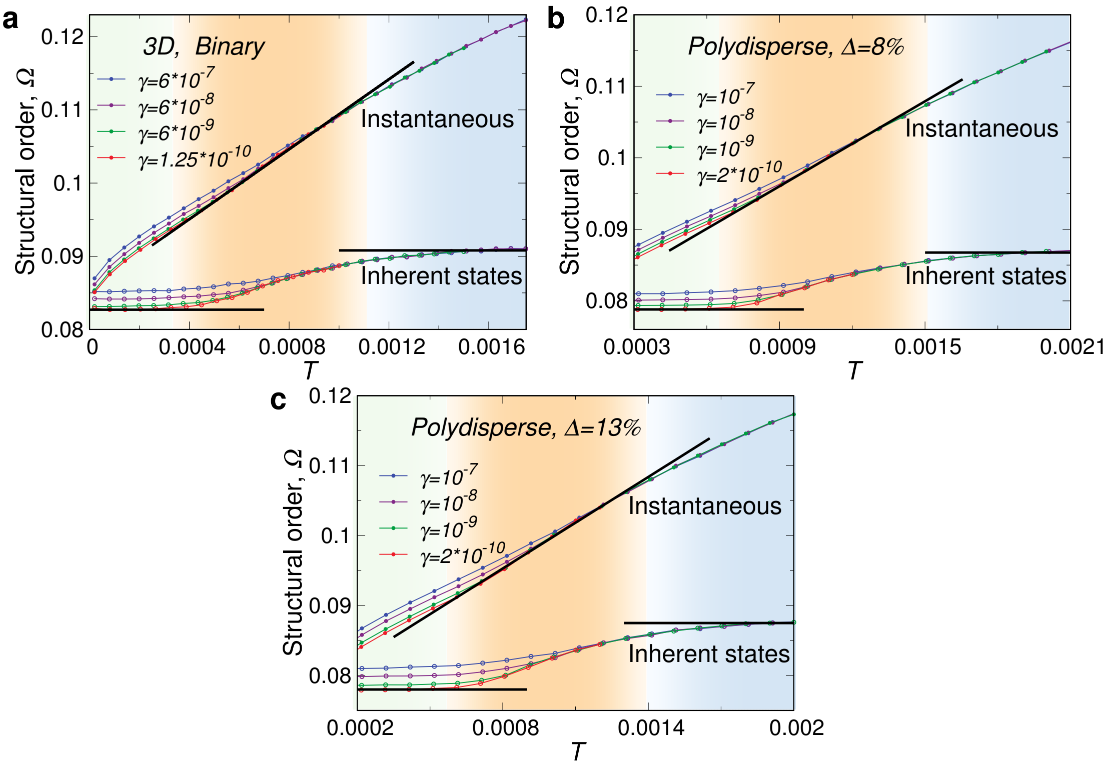

Supplementary Figure 6: **Structure formation during cooling in 3D harmonic systems.** Corresponding to Fig. 1a in the main text, evolution of structural order Ω in instantaneous (filled circles) and corresponding inherent states (open circles) in 3D binary systems (**a**), and 3D polydisperse systems with $\Delta = 8\%$ (**b**) and $\Delta = 13\%$ (**c**). Data are shown for different cooling rate γ. For instantaneous states, the temperature dependence of Ω can be fitted with a linear function in the supercooled regime: $(\Omega - \Omega_0)/\Omega_0 = \kappa(T - T_0)/T_0$ (solid line). For inherent states, Ω stays constant in both simple-liquid and glass regimes (horizontal lines).

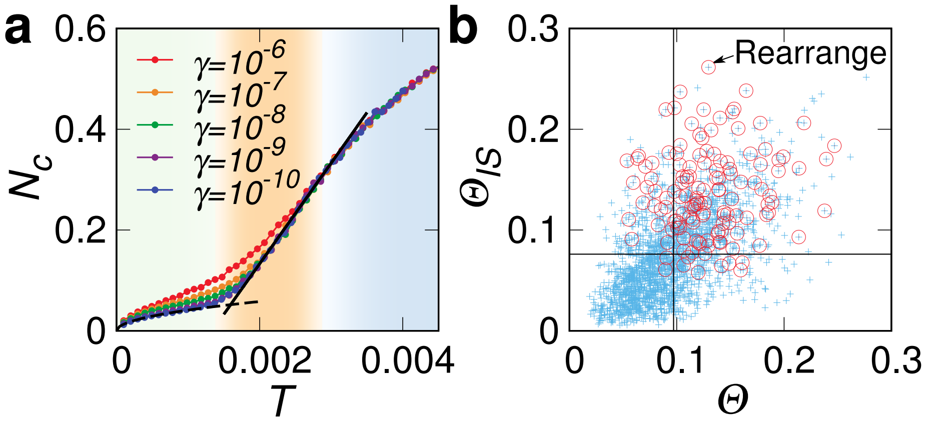

Supplementary Figure 7: **Correspondence of structures between instantaneous and inherent states in harmonic systems.** For a 2D polydisperse system ($\Delta = 13\%$) corresponding to Fig. 1 in the main text. **a,** Average number of neighbour change N_c between instantaneous and inherent ones. Dashed and solid lines are guides to the eye for different temperature dependences of N_c in the glass (green) and supercooled (orange) regimes, respectively. **b,** Scatter plot of structural order in instantaneous (Θ) and inherent states (Θ_{IS}) for a typical configuration at $T = 0.0015$ and $\gamma = 10^{-10}$. The particles which change neighbour when quenched into the inherent state from a finite temperature are indicated with red circles. The vertical and horizontal lines represent the average values of structural order in the instantaneous and inherent states, respectively.

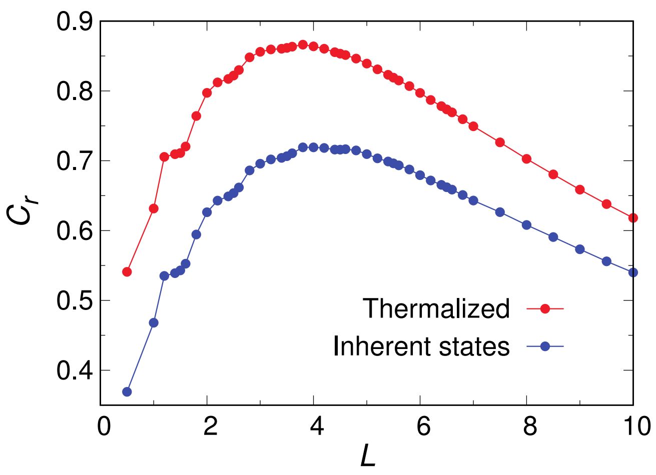

Supplementary Figure 8: **Comparison of structure-dynamics correlations based on instantaneous (thermalized) and inherent states.** For 2D polydisperse system ($\Delta = 13\%$) at $T = 0.0018$, we characterize the structural order in thermalized states and the corresponding inherent states, and then quantify the structure-dynamics correlation respectively. We find that the peak correlation is increased by 21.2% and reaches $C_r = 87\%$ by starting from thermalized states, which is unprecedented in previous studies.

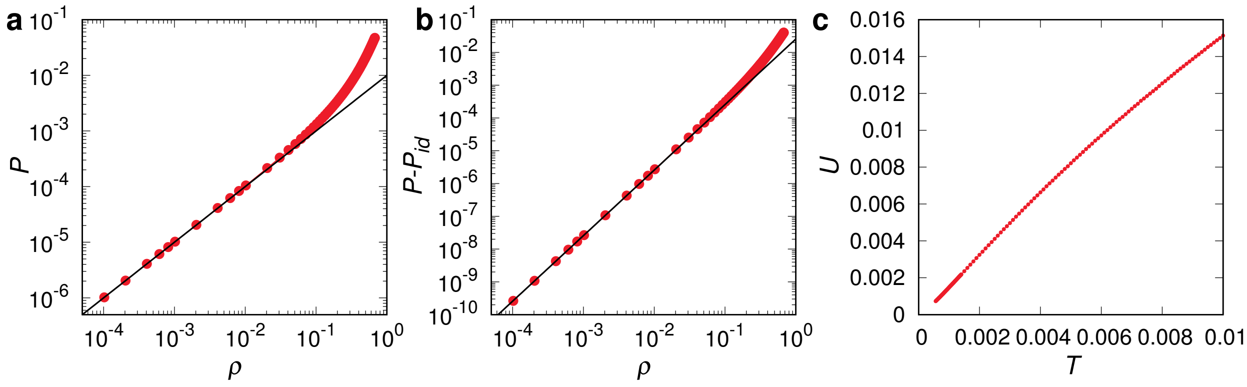

Supplementary Figure 9: **Equation of state in 3D harmonic systems.** We calculate the total entropy through thermodynamic integration starting from the low-density ideal-gas limit ($T = 0.01, \rho \to 0$) along the isotherm $T = 0.01$, up to the density studied $\rho = 0.675$. **a** and **b**, Density dependence of pressure P and the excess pressure over the ideal-gas value $P_{\rm id}$, respectively. The solid lines indicate the ideal-gas law $P \sim \rho$ and the predicted virial correction $P - P_{\rm id} \sim \rho^2$ in **a** and **b**, respectively. The good consistence between our data and the theoretical prediction suggests that we have properly accessed the ideal-gas limit. **c**, Temperature dependence of the potential energy U at fixed density $\rho = 0.675$. This is used for the calculation of the total entropy at target temperatures [10–13].

4. Relation between configurational entropy and our structural order parameter. Here we explore the relation between configurational entropy $S_{\rm conf}$ and our structural order parameters. In particular, we focus on binary glass formers to avoid possible issues related to the polydisperse distribution of particle sizes [21–23], the resolution of which is beyond the scope of the current work. $S_{\rm conf}$ under certain conditions is determined by the multiplicity of local potential energy minima sampled by the liquid. Following the well-established method in the literature [10–13], we evaluation $S_{\rm conf}$ by calculating the difference of the total entropy and the vibrational entropy of the basins

$$S_{\rm conf}(\rho, T) = S_{\rm tot}(\rho, T) - S_{\rm vib}(\rho, T). \qquad (2)$$

We calculate $S_{\rm tot}$ via thermodynamic integration starting from the ideal-gas reference point. For 3D harmonic systems under study, we first integrate from the ideal-gas limit ($\rho \to 0, T = 0.01$) to the reference state ($\rho = 0.675, T = 0.01$) along the isotherm. The density dependence of pressure P shown in Supplementary Figs. 9a and 9b confirms that we have properly accessed the ideal-gas limit. The total entropy at the target temperature along the isochoric path can

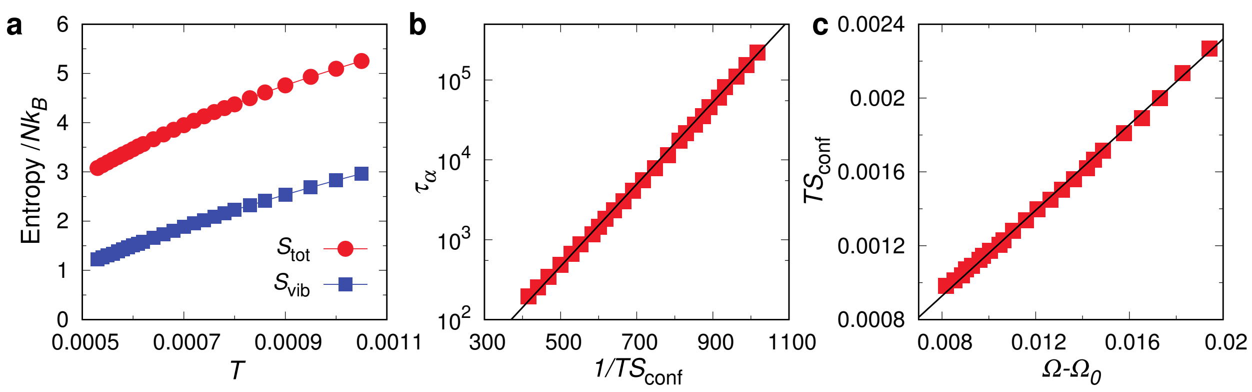

Supplementary Figure 10: **Entropy related analyses in 3D binary systems. a,** Temperature dependence of the total entropy $S_{\rm tot}$ (red circles) and vibrational entropy $S_{\rm vib}$ (blue squares). The configurational entropy is calculated from the following relation: $S_{\rm conf} = S_{\rm tot} - S_{\rm vib}$. **b,** Adam-Gibbs plot of structure relaxation time τ_α vs. $1/TS_{\rm conf}$. The data is well described by the Adam-Gibbs relation $\tau_\alpha = \tau_0 \exp(A/TS_{\rm conf})$, as indicated by the solid line. **c,** Relation between our structural order parameter and configurational entropy. The solid line suggests a linear relation $TS_{\rm conf} \sim \Omega - \Omega_0$.

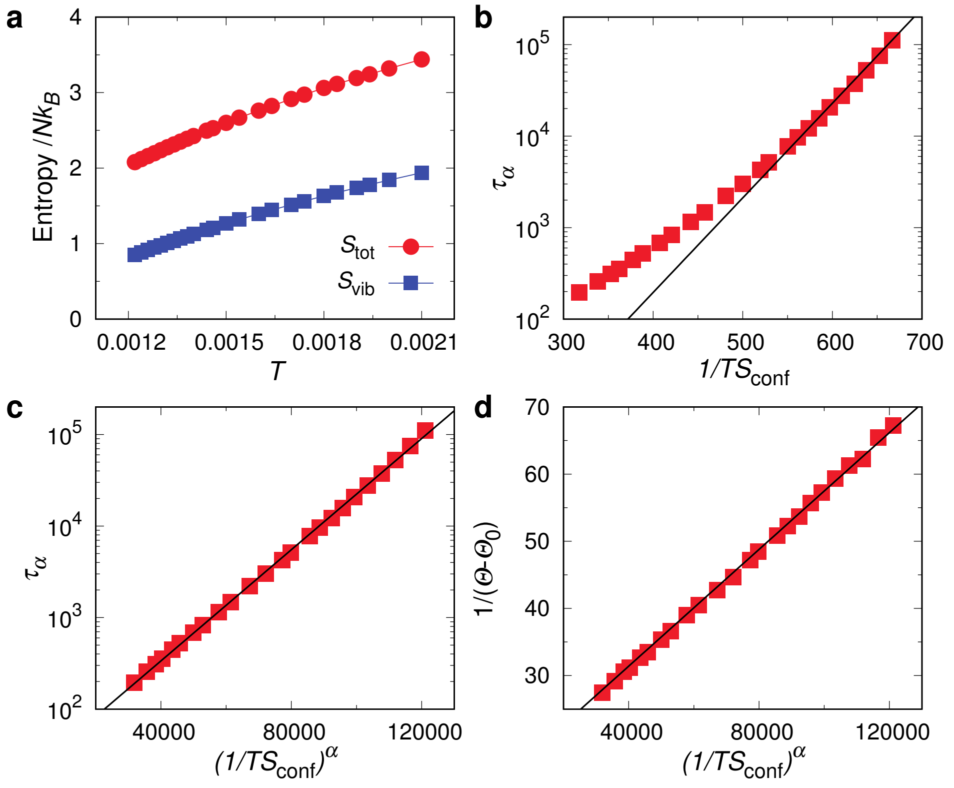

Supplementary Figure 11: **Entropy related analyses in 2D binary systems. a,** Temperature dependence of the total entropy $S_{\rm tot}$ (red circles) and the vibrational entropy $S_{\rm vib}$ (blue squares). The configurational entropy is calculated from the following relation: $S_{\rm conf} = S_{\rm tot} - S_{\rm vib}$. **b,** Adam-Gibbs plot of structure relaxation time τ_α vs. $1/TS_{\rm conf}$. The data is clearly deviates from the standard Adam-Gibbs relation $\tau_\alpha = \tau_0 \exp(A/TS_{\rm conf})$, as indicated by the solid line. **c,** Generalized Adam-Gibbs plot of structure relaxation time τ_α vs. $(1/TS_{\rm conf})^\alpha$, with $\alpha = 1.8$. The solid line indicate the generalized Adam-Gibbs relation $\tau_\alpha = \tau_0 \exp[(A/TS_{\rm conf})^\alpha]$. This observation is consistent with the previous studies in 2D [14]. **d,** Following the generalized Adam-Gibbs relation, the structural order parameter $1/(\Theta - \Theta_0)$ is plotted as a function of $(1/TS_{\rm conf})^\alpha$, with the same α as **c**. The solid line suggests a linear relation between them.

then be calculated using the temperature dependence of the potential energy U shown in Supplementary Figure 9c. For all results presented in the following, the vibrational entropy is calculated by approximating each basin as a harmonic well [10–13, 24]. This approach has been confirmed to be valid at low temperatures [10–13, 24], which agrees reasonably well with other methods [12, 23]. We refer to Refs. 10–13 for the detailed formulism.

Supplementary Figure 10a shows the temperature dependence of $S_{\rm tot}$ and $S_{\rm vib}$ in 3D binary systems, from which we calculate $S_{\rm conf}$ according to Eq. (2). Supplementary Figure 10b shows the Adam-Gibbs plot of structure relaxation time τ_α as a function of $1/TS_{\rm conf}$, which confirms the Adam-Gibbs relation $\tau_\alpha = \tau_0 \exp(A/TS_{\rm conf})$ in this system [14, 24, 25]. In comparison with Eq. (2) in the main text, this result motivates a direct relation between our structural order parameter and configurational entropy. This point is confirmed in Supplementary Figure 10c.

We further explore the relation between $S_{\rm conf}$ and our structural order parameter Θ in 2D binary systems. Previously, it has been shown that τ_α does not following the standard Adam-Gibbs relation in 2D, but rather a generalized Adam-Gibbs relation $\tau_\alpha = \tau_0 \exp[(A/TS_{\rm conf})^\alpha]$ with $\alpha \neq 1$ [14]. As shown in Supplementary Figs. 11a-c, our results are also described by the generalized form of Adam-Gibbs relation. Accordingly, we find a linear relation between our structural order parameter $1/(\Theta - \Theta_0)$ and $(1/TS_{\rm conf})^\alpha$. Although a fundamental understanding of such a relation lacks at present, which is interesting to be explored further [26], this result nevertheless point to an intriguing connection between structural order as measured by our order parameter and configurational entropy.

5. Relation between Θ and local structure entropy. Here we study the local structure entropy s_2 at a particle level, which is a local version of the two-body translational correlation contribution to the excess entropy [27]:

$$s_{2,i} = -\frac{k_B \rho}{2} \int d\mathbf{r} \left\{ g_i(\mathbf{r}) \ln g_i(\mathbf{r}) - [g_i(\mathbf{r}) - 1] \right\}, \quad (3)$$

where k_B is the Boltzmann constant, ρ is the number density, and $g_i(\mathbf{r})$ is the pair correlation function between particle i and the other particles. For simplicity, we focus on a 2D polydisperse system ($\Delta = 13\%$), but generality is also expected for other systems. Supplementary Figure 12a shows the spatial distribution of s_2, which is highly correlated with that of bare Θ (see Fig. 3d in the main text). Such a direct correlation can be more easily visualized after spatial coarse-graining of both quantities, as shown in Supplementary Figure 12b for $s_{2,\rm CG}$ and Fig. 3e in the main text for $\Theta_{\rm CG}$. This result indicates that similar to hard-sphere crystallization, sterically favoured structures sacrifice configurational entropy to gain more vibrational entropy and thus low free energy locally. This supports free-energy-driven formation of locally favoured structures [28]. The temperature dependence of $P(s_2)$ is shown in Supplementary Figure 12c. A shift to a lower value of s_2 is seen with decreasing temperature, indicating a growth of structural correlation. From the inset, we can see that ordered particles contribute to the development of the tail of s_2 towards a negative direction. At the same time, we note that local structure entropy is not local in a strict sense

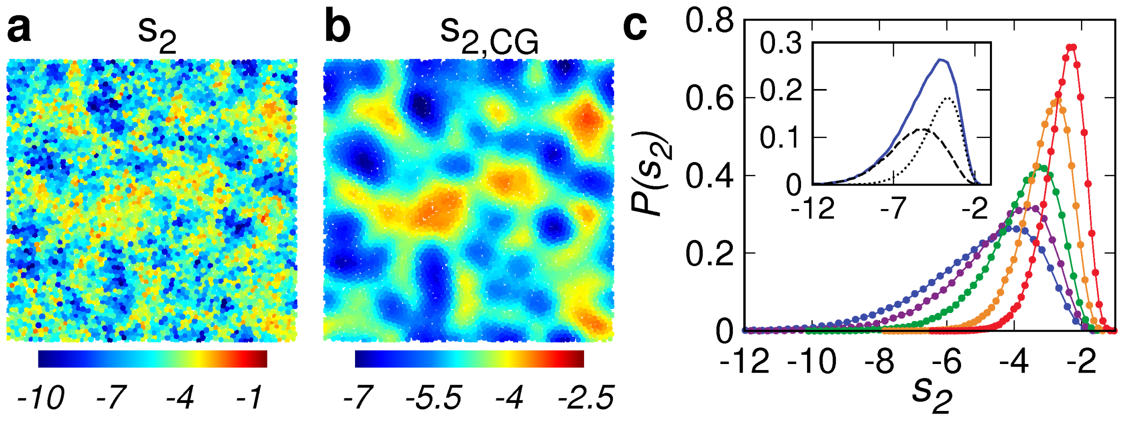

Supplementary Figure 12: **Local structure entropy. a,b,** Spatial distributions of bare two-body excess entropy s_2 and that coarse-grained at $L = 3.8$, $s_{2,\rm CG}$, respectively, for a 2D PM ($\Delta = 13\%$) at $T = 0.0018$. See the correspondence between these two panels and Figs. 3c-e in the main text. **c,** Probability distribution of s_2 for a range of temperatures (decreasing temperature from right to left, with $T = 0.0037$, 0.0028, 0.0023, 0.002, and 0.0018). Inset: For $T = 0.0018$, probability distribution of s_2, $P(s_2)$, for the whole system (blue solid), half particles with smallest Θ hence most ordered (black dashed), and the other half particles with largest Θ hence most disordered (black dotted).

(by taking the upper integration limit of Eq. (3) to long-distance). This makes a quantitative comparison between s_2 and our order parameter illusive.

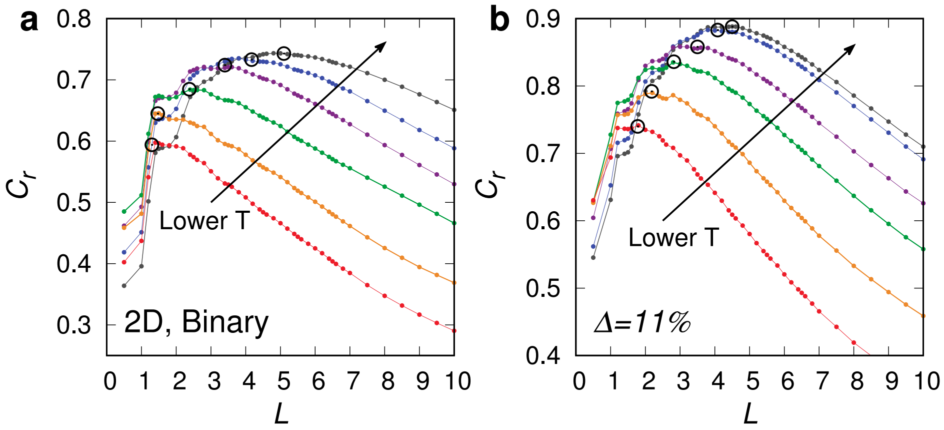

Supplementary Figure 13: **Nonlocal correlation between structural order and microscopic relaxation in 2D harmonic systems.** Corresponding to Fig. 3b in the main text, correlation between microscopic relaxation time (τ_α for each particle) and structural order as functions of coarse graining length L in 2D binary systems (**a**) and 2D polydisperse systems ($\Delta = 11\%$) (**b**). As indicated by the arrows, different curves correspond to decreasing temperatures of $T = 0.0031$, 0.0024, 0.002, 0.0017, 0.0015, and 0.00135 in **a** and $T = 0.0038$, 0.003, 0.0026, 0.0023, 0.002, and 0.0019 in **b**. The peak positions are indicated by black circles.

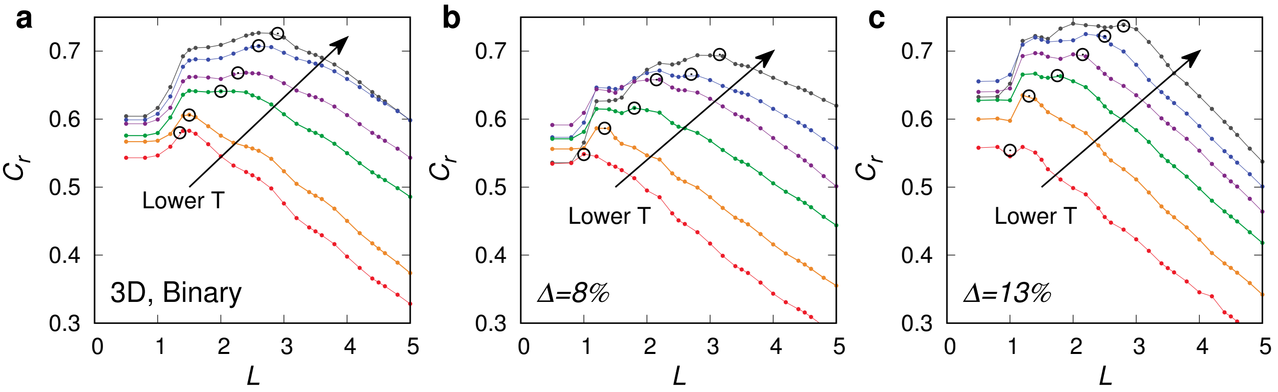

Supplementary Figure 14: **Nonlocal correlation between structural order and microscopic relaxation in 3D harmonic systems.** Corresponding to Fig. 3b in the main text, correlation between microscopic relaxation time (τ_α for each particle) and structural order as functions of coarse graining length L in 3D binary systems (**a**), and 3D polydisperse systems with $\Delta = 8\%$ (**b**) and $\Delta = 8\%$ (**c**). As indicated by the arrows, different curves correspond to decreasing temperatures of $T = 0.0012$, 0.001, 0.0008, 0.0007, 0.0006, and 0.000556 in **a**, $T = 0.0016$, 0.00125, 0.0011, 0.001, 0.0009, and 0.0008 in **b**, and $T = 0.00135$, 0.0011, 0.001, 0.0009, 0.00082, and 0.000763 in **c**. The peak positions are indicated by black circles.

6. Nonlocal scenario for structure relaxation. Corresponding to Fig. 3b in the main text, in Supplementary Figs. 13 and 14, we show the correlation between the microscopic relaxation time (τ_α for each particle) and the structural order as functions of the coarse-graining length L for 2D and 3D systems, respectively. We generally observe that C_r significantly increases initially with increasing the coarse-graining length and maximizes around a temperature-dependent length scale, which is identified as the characteristic static correlation length ξ of the underlying structure. We notice from a close inspection of the correlation curves $C_r(L)$ that they are quite smooth in 2D while more fluctuated in 3D. We speculate that this is due to the large number of particles in each neighbour shell in 3D, and hence a significant influence from the density fluctuations when the coarse-graining length covers an additional neighbour shell. An improved version of coarse-graining might be necessary to smooth out such fluctuations.

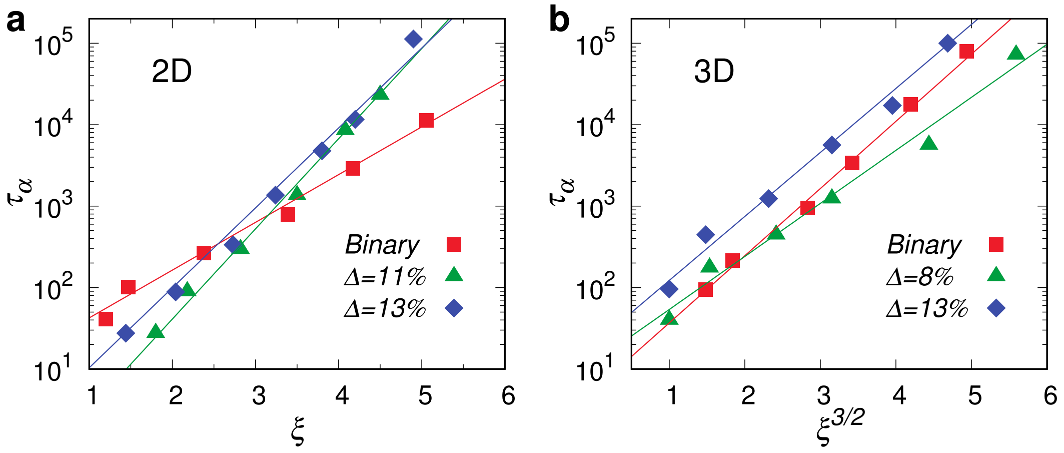

Supplementary Figure 15: **Relationship between structure relaxation time and static correlation length in harmonic systems.** Relationship between τ_α and ξ for 2D disk systems (**a**) and 3D sphere systems (**b**). The solid lines show the relation $\tau_\alpha = \tau_0 \exp[D(\xi/\xi_0)^{d/2}]$, where d is the spatial dimension.

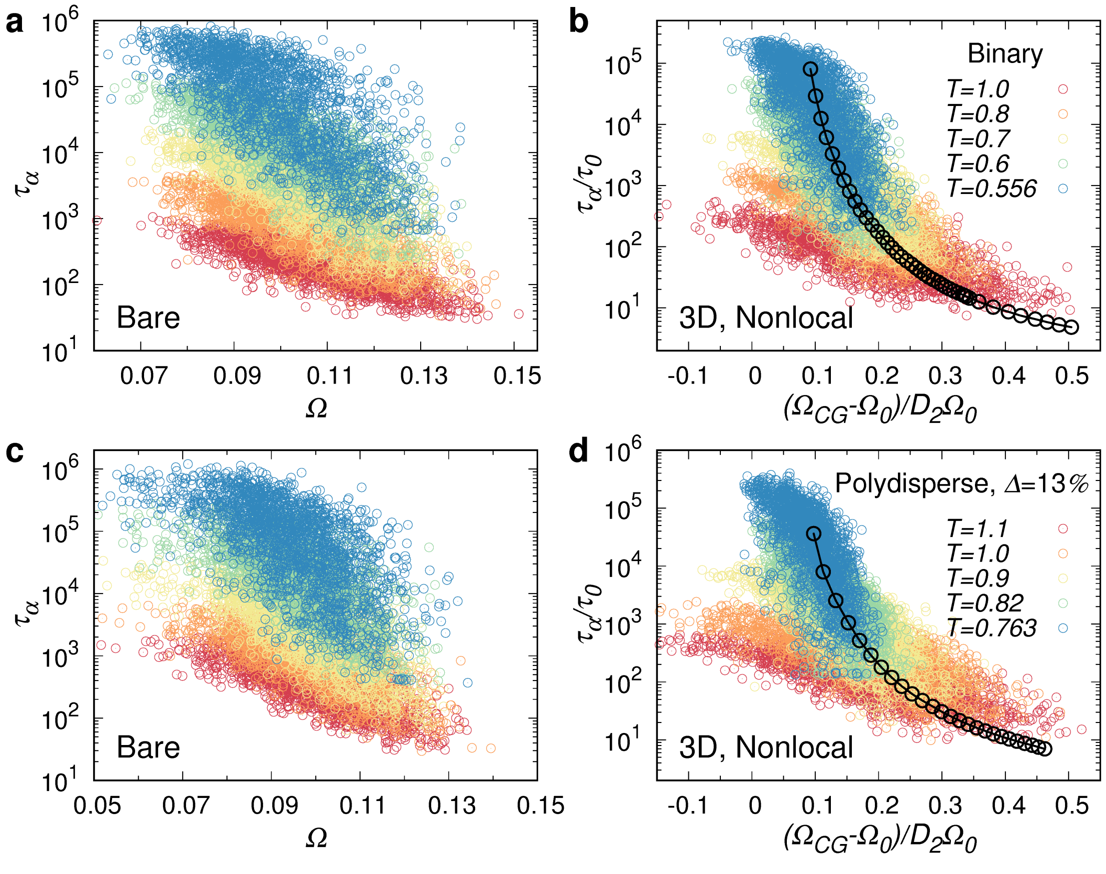

Supplementary Figure 16: **Microscopic relation between structural order and relaxation in 3D harmonic systems.** The results shown here correspond to Figs. 4c and 4d in the main text. **a,** Microscopic relaxation time τ_α as a function of coarse grained structural order $\Omega_{\rm CG} - \Omega_0$ for 3D binary systems at different temperatures (T is given in the unit of 10^{-3} in the legends). On top of the scatter plots, the relation between macroscopic τ_α and global structural order are shown together. **b,** Corresponding to **a**, microscopic relaxation time τ_α as a function of bare structural order. **c** and **d,** The same analysis as in **a** and **b** for 3D polydisperse systems with $\Delta = 13\%$.

7. Relationship between structure relaxation time τ_α and static correlation length ξ. An important question concerning the nature of glass transition is whether the drastic dynamical slowing down originates from the growth of the underlying static order. In Supplementary Figure 15, we show the relation between structure relaxation time τ_α and the static correlation length ξ for both 2D and 3D systems. In general, the relation $\tau_\alpha = \tau_0 \exp[D(\xi/\xi_0)^{d/2}]$ with d being the spatial dimension gives a reasonable description of the data, suggesting a common structural origin of slow glassy dynamics. This observation is consistent with previous studies [27, 28] and can be rationalized in an Ising-type critical scenario for glass transition [27–29]. We note that this relation is also consistent with the RFOT scenario [30–32], although such a relation is usually expected for below $T_{\rm mct}$ in that scenario.

8. Microscopic relation between structural order and relaxation in 3D. Corresponding to Figs. 4c and 4d in the main text, in Supplementary Figure 16, we show how the microscopic structure relaxation is related to the structural order in 3D binary and polydisperse ($\Delta = 13\%$) systems. The same behaviours as the 3D polydisperse systems with $\Delta = 8\%$ are observed that, after coarse-graining, the microscopic relation between structural order and relaxation tends to follow the macroscopic one in the case of deep supercooling.

9. Intrinsic fluctuations of microscopic relaxation dynamics. In Supplementary Figure 16 and also Fig. 4 in the main text, we have shown the correspondence between single-particle structural order and microscopic relaxation time. Good correlations are reached at lowest temperatures (see also Supplementary Figs. 13 and 14), but certain degrees of fluctuations, meaning a certain width of scattering of the microscopic data points around the macroscopic relation, are seen. To understand the origin of such fluctuations, here we study the microscopic relaxation dynamics in more detail. In addition to the self-intermediate scattering function for the whole system and each particle defined through the isoconfigurational average (see Methods in the main text), we calculate the self-intermediate scattering function for each particle and in each run in the isoconfigurational ensemble. This additional analysis would provide further information about the intrinsic fluctuations of single-particle relaxation dynamics. For particle j in the $a_{\rm th}$ run, $F_s^{j,a}(k,t) = \cos\left(i\mathbf{k}\cdot[\mathbf{r}_j^a(t) - \mathbf{r}_j^a(0)]\right)$, with the superscript a indicating the $a_{\rm th}$ trajectory. Supplementary Figure 17 shows $F_s(k,t)$ averaged over the whole system (dark blue circles), for an immobile (green squares, left) or mobile (red squares, right) particle averaged within the isoconfigurational ensemble, and for that particle in different runs of the isoconfigurational ensemble (fluctuating curves in the background). Several interesting observations can be made from Supplementary Figure 17: (1) Not only that each particle may relax differently from the global average, but

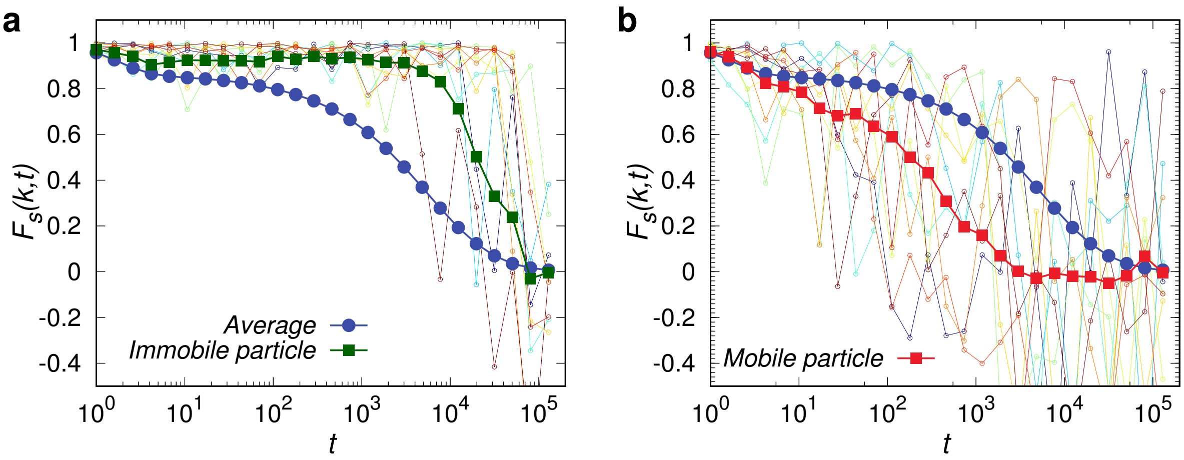

Supplementary Figure 17: **Intrinsic fluctuations of microscopic relaxation dynamics.** Self-intermediate scattering functions $F_s(k,t)$ for 2D polydisperse system ($\Delta = 13\%$) at $T = 0.0018$. **a,** $F_s(k,t)$ averaged over the whole system, for an immobile particle averaged within the isoconfigurational ensemble, and for that particle in different runs of the isoconfigurational ensemble (fluctuating curves in the background, 10 different realizations are shown). This immobile particle has a structural order $\Theta_{\rm Bare} = 0.07344$ and $\Theta_{\rm CG} = 0.09228$. **b,** The same plot as in **a** but with the selected particle being mobile. This mobile particle has a structural order $\Theta_{\rm Bare} = 0.1224$ and $\Theta_{\rm CG} = 0.1166$. Note that larger Θ means less ordered. We see that not only each particle may relax differently from the global average, but also the same particle with exactly the same initial static structural order may relax differently due to different initial velocity fields in the isoconfigurational ensemble. See text for more discussions.

also the same particle with exactly the same initial static structural order may relax very differently due to different initial velocity fields in the isoconfigurational ensemble. (2) The immobile particle has a high structural order, and its relaxation dynamics is more determinate. The structure relaxation is found to take place with a narrow time window in different runs. (3) In comparison, the mobile particle has a low structural order, and the relaxations significantly differ in different runs. This observation can be rationalized from the competition between structural order (determinate effect) and thermal fluctuation (stochastic effect). Thermal fluctuation plays an intrinsic role in the microscopic dynamics; therefore, in the case of weak local structural order, the particle tends to relax randomly. Consequently, the microscopic relaxation time that we measure for each particle should be understood in a statistical sense, which is intrinsically affected by the thermal fluctuations. Meanwhile, we should mention that our structural order parameters are adequate, but by no meaning, perfect and coarse-graining is a working but approximate procedure. These also contribute to the scattering of data points in Fig. 4 in the main text and Supplementary Figure 16, in addition to the intrinsic thermal fluctuations.

SUPPLEMENTARY REFERENCES

[1] Berendsen, H. J., Postma, J. v., van Gunsteren, W. F., DiNola, A. & Haak, J. Molecular dynamics with coupling to an external bath. *J. Chem. Phys.* **81**, 3684–3690 (1984).

[2] Allen, M. P. & Tildesley, D. J. *Computer simulation of liquids* (Oxford university press, 2017).

[3] Bannerman, M. N., Sargant, R. & Lue, L. Dynamo: a free o(n) general event-driven molecular dynamics simulator. *J. Comp. Chem* **32**, 3329–3338 (2011).

[4] Widmer-Cooper, A., Harrowell, P. & Fynewever, H. How reproducible are dynamic heterogeneities in a supercooled liquid? *Phys. Rev. Lett.* **93**, 135701 (2004).

[5] Widmer-Cooper, A. & Harrowell, P. Predicting the long-time dynamic heterogeneity in a supercooled liquid on the basis of short-time heterogeneities. *Phys. Rev. Lett.* **96**, 185701 (2006).

[6] Bitzek, E., Koskinen, P., Gähler, F., Moseler, M. & Gumbsch, P. Structural relaxation made simple. *Phys. Rev. Lett.* **97**, 170201 (2006).

[7] Berthier, L. & Tarjus, G. Nonperturbative effect of attractive forces in viscous liquids. *Phys. Rev. Lett.* **103**, 170601 (2009).

[8] Berthier, L. & Tarjus, G. The role of attractive forces in viscous liquids. *J. Chem. Phys.* **134**, 214503 (2011).

[9] Ingebrigtsen, T. S., Schrøder, T. B. & Dyre, J. C. What is a simple liquid? *Phys. Rev. X* **2**, 011011 (2012).

[10] Sciortino, F., Kob, W. & Tartaglia, P. Inherent structure entropy of supercooled liquids. *Phys. Rev. Lett.* **83**, 3214 (1999).

[11] Sciortino, F., Kob, W. & Tartaglia, P. Thermodynamics of supercooled liquids in the inherent-structure formalism: a case study. *J. Phys.: Condens. Matter* **12**, 6525 (2000).

[12] Sastry, S. Evaluation of the configurational entropy of a model liquid from computer simulations. *J. Phys.: Condens. Matter* **12**, 6515 (2000).

[13] Sastry, S. Liquid limits: Glass transition and liquid-gas spinodal boundaries of metastable liquids. *Phys. Rev. Lett.* **85**, 590 (2000).

[14] Sengupta, S., Karmakar, S., Dasgupta, C. & Sastry, S. Adam-gibbs relation for glass-forming liquids in two, three, and four dimensions. *Phys. Rev. Lett.* **109**, 095705 (2012).

[15] Shiba, H., Yamada, Y., Kawasaki, T. & Kim, K. Unveiling dimensionality dependence of glassy dynamics: 2D infinite fluctuation eclipses inherent structural relaxation. *Phys. Rev. Lett.* **117**, 245701 (2016).

[16] Illing, B. *et al.* Mermin–wagner fluctuations in 2D amorphous solids. *Proc. Natl Acad. Sci. USA* **114**, 1856–1861 (2017).

[17] Vivek, S., Kelleher, C. P., Chaikin, P. M. & Weeks, E. R. Long-wavelength fluctuations and the glass transition in two dimensions and three dimensions. *Proc. Natl Acad. Sci. USA* **114**, 1850–1855 (2017).

[18] Sastry, S., Debenedetti, P. G. & Stillinger, F. H. Signatures of distinct dynamical regimes in the energy landscape of a glass-forming liquid. *Nature* **393**, 554 (1998).

[19] Kob, W., Roldán-Vargas, S. & Berthier, L. Non-monotonic temperature evolution of dynamic correlations in glass-forming liquids. *Nat. Phys.* **8**, 164–167 (2012).

[20] Gellatly, B. J. & Finney, J. L. Characterisation of models of multicomponent amorphous metals: the radical alternative to the voronoi polyhedron. *J. Non-Cryst. Solids* **50**, 313–329 (1982).

[21] Ozawa, M. & Berthier, L. Does the configurational entropy of polydisperse particles exist? *J. Chem. Phys.* **146**, 014502 (2017).

[22] Baranau, V. & Tallarek, U. Another resolution of the configurational entropy paradox as applied to hard spheres. *J. Chem. Phys.* **147**, 224503 (2017).

[23] Ozawa, M., Parisi, G. & Berthier, L. Configurational entropy of polydisperse supercooled liquids. *J. Chem. Phys.* **149**, 154501 (2018).

[24] Sastry, S. The relationship between fragility, configurational entropy and the potential energy landscape of glass-forming liquids. *Nature* **409**, 164 (2001).

[25] Adam, G. & Gibbs, J. H. On the temperature dependence of cooperative relaxation properties in glass-forming liquids. *J. Chem. Phys.* **43**, 139–146 (1965).

[26] Ozawa, M., Scalliet, C., Ninarello, A. & Berthier, L. Does the adam-gibbs relation hold in simulated supercooled liquids? *J. Chem. Phys.* **151**, 084504 (2019).

[27] Tanaka, H., Kawasaki, T., Shintani, H. & Watanabe, K. Critical-like behaviour of glass-forming liquids. *Nat. Mater.* **9**, 324–331 (2010).

[28] Tanaka, H. Bond orientational order in liquids: Towards a unified description of water-like anomalies, liquid-liquid transition, glass transition, and crystallization. *Eur. Phys. J. E* **35**, 113 (2012).

[29] Langer, J. S. Ising model of a glass transition. *Phys. Rev. E* **88**, 012122 (2013).

[30] Kirkpatrick, T. R., Thirumalai, D. & Wolynes, P. G. Scaling concepts for the dynamics of viscous liquids near an ideal glassy state. *Phys. Rev. A* **40**, 1045–1054 (1989).

[31] Lubchenko, V. & Wolynes, P. G. Theory of structural glasses and supercooled liquids. *Annu. Rev. Phys. Chem.* **58**, 235–266 (2007).

[32] Berthier, L. & Biroli, G. Theoretical perspective on the glass transition and amorphous materials. *Rev. Mod. Phys.* **83**, 587–645 (2011).
